# PEG Middle-Block Molecular Weight as a Quantitative Design Variable for Stereocomplex Formation Efficiency in High-Molecular-Weight PLLA–PEG–PLLA/PDLA Blends

**DOI:** 10.1021/acsomega.6c08171

**Published:** 2026-09-25

**Authors:** Dutchanee Pholharn, Samaneh Dehghani, Onpreeya Veang-in, Yottha Srithep

**Affiliations:** † Department of Chemistry, Faculty of Science and Technology, 54783 Rajabhat Mahasarakham University, Mahasarakham 44000, Thailand; ‡ Department of Wind Energy, Kunsan National University, Gunsan 54150, Republic of Korea; § Department of Mechanical Engineering, Faculty of Industry and Technology, 202946 Rajamangala University of Technology Isan, Sakon Nakhon 47160, Thailand; ∥ Manufacturing and Materials Research Unit, Faculty of Engineering, Mahasarakham University, Mahasarakham 44150, Thailand

## Abstract

Achieving complete stereocomplex (SC) formation in high-molecular-weight PLLA/PDLA blends by injection molding remains a challenge because the rapid mold-cooling rate suppresses the slow enantiomeric chain-recognition process. Here, we demonstrate that the PEG middle-block molecular weight in PLLA–PEG–PLLA triblock copolymers is the decisive kinetic control variable governing SC formation efficiency (*f*_SC_). Copolymers with PEG *M*_n_ = 400, 4000, and 35,000 g/mol were synthesized by bulk ring-opening polymerization (ROP) at two PLLA-*block M*_n_ targets (100,000 and 200,000 g/mol) and blended 1:1 with PDLA before injection molding. *W*_PLA_-corrected differential scanning calorimetry (DSC) showed that *f*_SC_ increased monotonically from approximately 75–76% (PEG400) to 93–95% (PEG4k) and to 100% (PEG35k), where the value was confirmed independently by X-ray diffraction (XRD). PEG35k-containing SC specimens crystallized completely during mold cooling without any postcooling cold crystallization, establishing *M*_n,PEG_ ≥ 35,000 g/mol as the practical threshold for complete in-mold stereocomplex at *M*_n_ ≈ 200,000 g/mol without postprocessing annealing. Toughness increased 7.1-fold, from 10.1 MJ m^–3^ (neat PLLA) to 71.5 MJ m^–3^ (L100 K-35k-L100 K/PDLA), driven by synergistic PEG-induced ductility and SC crystal reinforcement. All SC–PLLA-PEG-PLLA specimens maintained dimensional stability at 200 °C, whereas non-SC counterparts melted.

## Graphical abstract

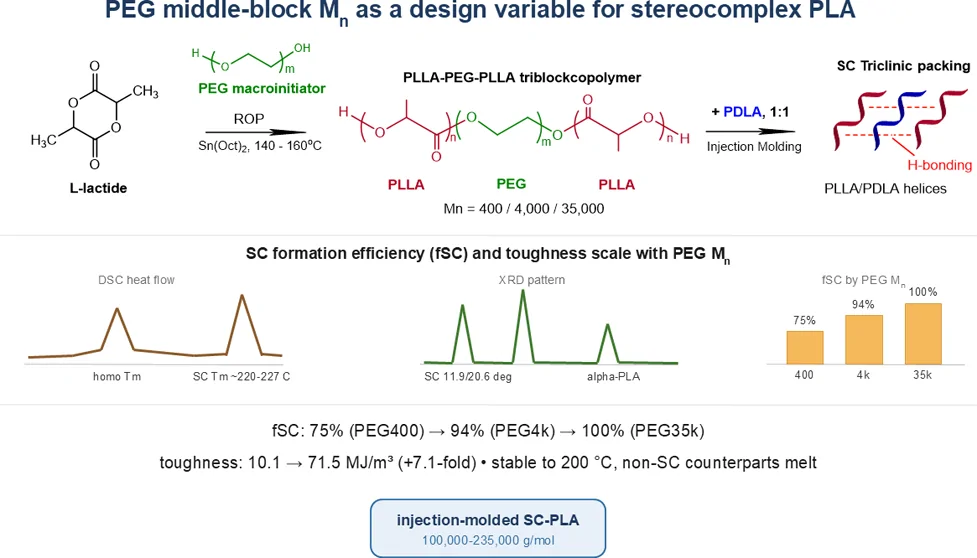

## 1 Introduction

Poly­(lactic acid) (PLA) has emerged as the primary biobased, compostable thermoplastic, with a production exceeding 400,000 t year^–1^ and applications including food packaging, single-use textiles, biomedical implants, and durable engineering components.^1–3^ Accelerating global restrictions on fossil-derived plastics have amplified demand for high-performance PLA grades that can compete with polymers such as poly­(ethylene terephthalate) and polypropylene. The principal obstacles are a glass transition temperature (*T*_g_ ≈ 55–60 °C), a heat-distortion temperature below 60 °C, slow crystallization kinetics, and brittleness.^4–6^

Stereocomplex (SC) crystallization between enantiomeric poly­(l-lactic acid) (PLLA) and poly­(d-lactic acid) (PDLA) addresses these limitations simultaneously.^7^ Discovered by Ikada et al.,^8^ SC crystals adopt a triclinic unit cell with a helical conformation stabilized by dense intermolecular C–H···O=C hydrogen bonds. The resulting melting temperature (*T*_m_ ≈ 220–230 °C) is 50–60 °C above that of homocrystalline PLA, and the crystallite network simultaneously increases tensile modulus, toughness, hydrolytic resistance, and gas-barrier performance.^9,10^ Despite these compelling properties, translation to industrial melt processing has been impeded by a well-known molecular weight: Tsuji and Ikada showed that melt-blended PLLA/PDLA enantiomers form SC crystals selectively only when the weight-average molecular weight *M*_w_ of each component is below approximately 60,000–100,000 g/mol; above this threshold, increased chain entanglement density and reduced diffusivity preferentially drive homocrystallization over stereocomplexation.^11–13^ Recent work has further demonstrated that stereocomplex crystallization in high-molar-mass enantiomeric PLA blends can be significantly enhanced through the incorporation of double-grafted copolymers.^14^ Molecular-weight-dependent stereocomplex crystallization has also recently been demonstrated in modified PLLA/PDLA blend systems.^15^ Na et al. further clarified that SC formation in high-*M*_w_ proceeds through melting and recrystallization of preformed homocrystals.^16^ Recent studies have further elucidated stereocomplex formation through homorecrystallization and mesophase-growth mechanisms.^17^ Strategies reported to extend the SC-forming range include nucleating agents,^18^ solvent-assisted processing,^19^ multifunctional chain extenders,^20^ and ABA-type block copolymer architectures.^21,22^

Among these, the incorporation of poly­(ethylene glycol) (PEG) as a middle block in PLLA–PEG-PLLA triblock copolymers has attracted sustained interest because PEG simultaneously plasticizes the PLLA matrix, enhances chain-segment mobility, and acts as a soft crystallizable block capable of nucleating PLA crystallization at phase-separated PEG domains.^23,24^ Li et al. demonstrated that PLLA–PEG–PLLA triblocks function as macromolecular plasticizers that accelerate PLLA crystallization from the melt.^25^ Bikiaris et al. reported that longer PEG blocks in PLA-*b*-PEG-*b*-PLA triblocks decrease *T*_g_ by up to 15 K and accelerate segmental relaxation dynamics.^26^ In the context of SC crystallization, Song et al. showed that a PEG segment in PDLA-*b*-PEG-*b*-PDLA promotes enantiomeric chain recognition in blends with PLLA;^27^ Han et al. demonstrated that raising the PEG molecular weight to 4000 g/mol in PLLA/PEG triblock blends with PDLA improves both SC crystal content and thermomechanical properties;^28^ and Xie et al. applied XRD Gaussian peak deconvolution to quantify SC and homocrystal fractions in self-assembled PLLA–PEG-PLLA/PDLA–PEG-PDLA aqueous systems.^29^ Nonisothermal crystallization behavior of PLLA/PDLA/PEG blends has been explored,^30^ and solvent-cast PDLA-*b*-PEG-*b*-PDLA/PLLA composites were reported by Liu et al.^31^ Recent work has also demonstrated the feasibility of producing heat-resistant PLA by injection molding within the melting range of preformed stereocomplex crystals.^32^

Despite these advances, critical gaps remain. Prior systematic studies employed relatively short PLA blocks (*M*_n_ ≤ 50,000 g/mol); the behavior of high-*M*_n_ PLLA–PEG–PLLA triblocks (up to 200,000 g/mol) under the rapid cooling conditions of industrial injection molding has not been characterized. Han et al.^28^ extended PEG *M*_n_ to 4000 g/mol; the impact of a wider PEG range extending to 35,000 g/mol on SC crystallization, mechanical performance, and heat resistance in injection-molded specimens is unexplored.

The present study addresses gaps by investigating PEG middle-block *M*_n_ (400, 4000, and 35,000 g/mol) as a quantitative design variable governing SC crystallization efficiency, thermal properties, crystalline microstructure, mechanical performance, and dimensional heat stability of injection-molded PLLA–PEG–PLLA/PDLA blends at two *M*_n_ targets (100,000 and 200,000 g/mol). An important and often overlooked processing prerequisite, the minimum molecular weight threshold for successful injection molding of SC blends, is also established. Furthermore, solution-cast SC film data are presented as reference benchmarks to quantify the crystallization deficit imposed by the rapid mold-cooling inherent to injection processing. Analytical contributions include: (i) *W*_PLA_-corrected crystallinity that accounts for the PEG mass fraction; (ii) *f*_SC_ as a single comparative metric; (iii) XRD peak deconvolution for independent crystallinity quantification; (iv) toughness from the triangular stress–strain approximation; and (v) cold crystallization kinetics analysis. The findings provide quantitative design guidelines for developing high-performance, melt-processable SC–PLA materials.

## 2 Experimental Section

### 2.1 Materials

l-Lactide (purity ≥ 99.5%) was obtained from Total Corbion PLA Ltd. (Gorinchem, The Netherlands) and purified immediately before use by double recrystallization from ethyl acetate (70–80 °C) followed by vacuum drying at 50 °C for ≥ 24 h. Poly­(d-lactic acid) (PDLA, grade L120, *M*_n_ = 90,000 g/mol, *M*_w_ = 152,000 g/mol, PDI = 1.69) pellets were also obtained from Total Corbion PLA Ltd. Polyethylene glycol (PEG) with molecular weights of 400, 4000, and 35,000 g/mol (denoted PEG400, PEG4k, and PEG35k, respectively), 1-dodecanol (98%), stannous 2-ethylhexanoate (Sn­(Oct)_2_, 92.5–95%), and ethyl acetate (99.5%) were purchased from Sigma-Aldrich (St. Louis, MO, USA).

### 2.2 Synthesis of PLLA and PLLA–PEG–PLLA Triblock Copolymers

PLLA homopolymers and PLLA–PEG–PLLA triblock copolymers were prepared by bulk ring-opening polymerization (ROP) of l-lactide using Sn­(Oct)_2_ as catalyst. For PLLA homopolymers, 1-dodecanol served as the monofunctional initiator; for triblock synthesis, dihydroxyl-terminated PEG (PEG400, PEG4k, or PEG35k) acted as the bifunctional macroinitiator (Figure 1). Sample codes, molar feed ratios, target *M*_n_, and blends are compiled in Table 1.

**Figure 1:**
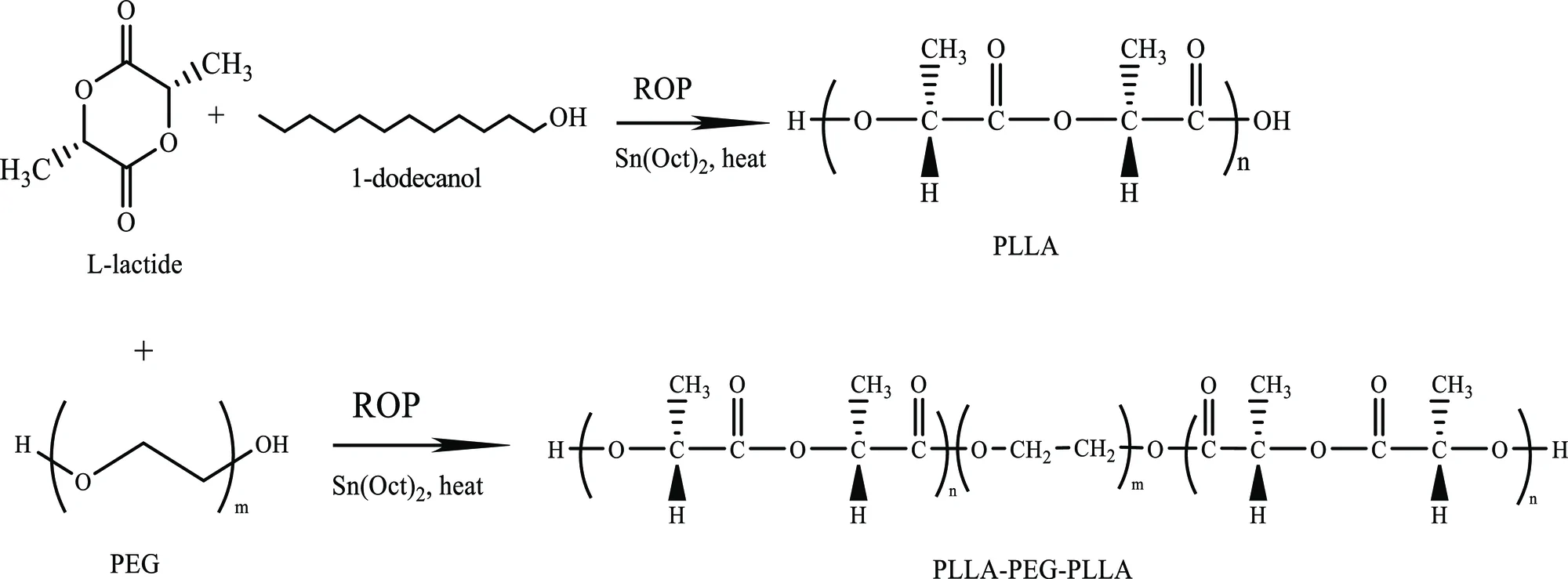
l-Lactide, PEG macroinitiators, Sn­(Oct)_2_ catalyst, and PDLA are processed by bulk ROP.

**Table 1:** Molecular Weights of Samples Synthesized via Ring-Opening Polymerization and Stereocomplex PLA Ratios

sample code	initiator	init. *M*_n_ (g/mol)	[L-LA]/[I]	*M*_n_ (g/mol)	SC blends	blend ratio (w/w)
L100 K	1-dodecanol	186	700:1	100,000	L100 K/PDLA	1:1
L200 K	1-dodecanol	186	1400:1	200,000	L200 K/PDLA	1:1
L50K-400-L50K	PEG400	400	700:1	100,400	L50K-400-L50K/PDLA	1:1
L100 K-400-L100 K	PEG400	400	1400:1	200,400	L100 K-400-L100 K/PDLA	1:1
L50K-4k-L50K	PEG4k	4000	700:1	104,000	L50K-4k-L50K/PDLA	1:1
L100 K-4k-L100 K	PEG4k	4000	1400:1	204,000	L100 K-4k-L100 K/PDLA	1:1
L50K-35k-L50K	PEG35k	35,000	700:1	135,000	L50K-35k-L50K/PDLA	1:1
L100 K-35k-L100 K	PEG35k	35,000	1400:1	235,000	L100 K-35k-L100 K/PDLA	1:1

In each batch, purified l-lactide (500 g) was charged with the appropriate initiator and Sn­(Oct)_2_ into a sealed, nitrogen-purged glass reactor and heated with continuous mechanical stirring for 4 h. Three macroinitiator-specific adaptations were required. (i) PEG400, which is a viscous liquid at room temperature, required a 10-fold reduction in catalyst loading to 0.01 mol % (relative to lactide) to prevent exothermic arising from instantaneous miscibility with the molten monomer. (ii) PEG35k, a waxy solid (*T*_m_ ≈ 65 °C), required polymerization at 140 °C rather than the standard 160 °C to ensure uniform macroinitiator melting prior to initiation and to prevent thermal degradation from localized overheating. (iii) PLLA homopolymers and PEG4k-initiated copolymers were polymerized under standard conditions (0.1 mol % Sn­(Oct)_2_, 160 °C). All reactions achieved 98–99% monomer conversion (Table 2). The product was dissolved in chloroform, precipitated into cold methanol, filtered, and dried under vacuum at 40 °C for 48 h. Full ^1^H NMR spectra confirming triblock architecture are provided in the Supporting Information (Section S1 and Figures S1–S8).

**Table 2:** Ring-Opening Polymerization Conditions and Molecular-Weight Characterization of Synthesized Polymers

sample	init. State	temp (°C)	time (h)	Sn(Oct)_2_ (mol %)	conv. (%)	*M*_n,GPC_ (g/mol)	*M*_w,GPC_ (g/mol)	PDI	*M*_n,NMR_ (g/mol)
L100 K	solid	160	4	0.1	99	53,700	90,100	1.60^a^	—
L200 K	solid	160	4	0.1	99	160,000	180,000	1.12	—
L50K-400-L50K	liquid	160	4	0.01	99	60,600	100,121	1.65^a^	87,000
L100 K-400-L100 K	liquid	160	4	0.01	99	178,000	190,200	1.10	160,000
L50K-4k-L50K	solid	160	4	0.1	99	48,000	67,000	1.40^a^	74,000
L100 K-4k-L100 K	solid	160	4	0.1	99	170,000	195,000	1.15	174,000
L50K-35k-L50K	solid	140	4	0.1	98	75,200	95,000	1.26	96,000
L100 K-35k-L100 K	solid	140	4	0.1	98	180,000	196,000	1.10	205,000

a PDI > 1.30 observed for the lower-*M*_n_ series (L100 K, L50K-400-L50K, L50K-4k-L50K) and attributed to broadened initiation kinetics at [L-LA]/[I] = 700:1; the elevated PDI for L50K-400-L50K (1.65) additionally reflects fast, exothermic initiation by the liquid PEG400 macroinitiator even at reduced catalyst loading.

### 2.3 Film-Forming and Injection-Mold Processability

Each polymer was subjected to a rapid film-forming screen prior to melt processing: 1 g was dissolved in 10 mL chloroform, cast into a glass Petri dish, and dried at ambient temperature until a free-standing film was obtained. Successful film formation, a continuous, self-supporting membrane without cracks or disintegration, confirms that *M*_n_ has exceeded the minimum chain-entanglement threshold for cohesive film behavior.^33^ Polymers with *M*_n_ < 80,000 g/mol (GPC) consistently failed to produce dimensionally stable injection-molded tensile bars, establishing 80,000 g/mol as the empirical *M*_n_ minimum for melt-injection processing of SC-PLA blends.

### 2.4 Preparation of Injection-Molded SC Specimens and Solution-Cast Films

Prior to melt processing, all PLLA, PLLA–PEG–PLLA, and PDLA materials were dried at 40 °C overnight to minimize residual moisture and potential hydrolytic degradation. Each PLLA or PLLA–PEG-PLLA polymer was ground into pellets and dry-blended with PDLA at 1:1 mass ratio (500 g each; Table 1). Blends were injection-molded into ASTM D638 Type I tensile bars using an INJ-58T reciprocating-screw machine (Charoen Tut Co., Ltd., Thailand) at a barrel temperature of 210 °C, mold temperature ≈ 25 °C, and injection pressure of 100 MPa. Cooling time was 30 s. Solution-cast SC reference films were prepared in parallel by codissolving each PLLA-component and PDLA (1:1 by mass, 0.5 g each) in 10 mL chloroform and casting into Petri dishes at ambient laboratory temperature and uncontrolled relative humidity. Slow solvent evaporation over 12–24 h provided a reference condition for comparison with the rapid cooling experienced during injection molding. Full DSC data for solution-cast films are reported in Table S1 of the Supporting Information.

### 2.5 Characterization

#### 2.5.1 Structural Characterization by ^1^H NMR Spectroscopy

Polymer microstructures were confirmed by ^1^H NMR spectroscopy (Varian, 400 MHz) using CDCl_3_ as solvent. Five resonances were used for structural assignment: PLLA methine (δ = 5.16 ppm, peak a), PLLA methyl (δ = 1.59 ppm, peak b), PEG methylene – OCH_2_CH_2_O– (δ = 3.65 ppm, peak c), PEG–ester linkage α-methylene (δ = 4.25 ppm, peak d), and terminal lactyl methyl (δ = 1.25 ppm, peak e).^34,35^ The c/a integral ratio yielded an independent estimate of *M*_n_ (denoted *M*_n,NMR_) used to cross-validate GPC results.

#### 2.5.2 Molecular Weight Determination by GPC

Molecular-weight characterization was performed on the purified and vacuum-dried synthesized polymers prior to injection molding. *M*_n_, *M*_w_, and the dispersity index (PDI = *M*_w_/*M*_n_) were measured by GPC using a Shimadzu RID-10A system equipped with a Styragel HR5E column (7.8 × 300 mm, M = 2000–4,000,000 g/mol), THF as mobile phase (flow rate 1.0 mL min^–1^, 40 °C), and refractive-index detection. Molecular weights were calculated relative to narrow polystyrene (PS) standards. For PEG-containing copolymers, GPC values are systematically underestimated relative to true *M*_n_ because the compact coil dimensions of PLA–PEG chains in THF yield smaller hydrodynamic volumes than linear PS of equivalent molar mass.^36^

#### 2.5.3 Differential Scanning Calorimetry

Thermal transitions were measured with a PerkinElmer DSC 4000 calorimeter. Specimens (3–5 mg) sealed in aluminum pans were heated from 30 to 250 °C at 10 °C min^–1^ under 20 mL min^–1^ N_2_ flow. Glass-transition temperature (*T*_g_), cold-crystallization temperature (*T*_c_), homocrystalline melting temperature (*T*_m1_), SC melting temperature (*T*_m2_), and the enthalpies (Δ*H*_c_, Δ*H*_m1_, Δ*H*_m2_) were extracted. The homocrystallinity (χ_homo_) and SC crystallinity (χ_SC_) were calculated with explicit correction for the PLA weight fraction (*W*_PLA_)­

1 $$\chi_{homo}\;\left(\%\right)=\left({\Delta H}_{homo}-{\Delta H}_{c}\right)/\left(93\;Jg^{-1}\times W_{PLA}\right)\times 100$$

2 $$\chi_{SC}\;\left(\%\right)={\Delta H}_{SC}/\left(142\;Jg^{-1}\times W_{PLA}\right)\times 100$$

Here 93 and 142 J g^–1^ are the theoretical melting enthalpies of 100% crystalline homocrystalline PLA and SC–PLA, respectively.^7,9^ *W*_PLA_ = mass fraction of total PLA (PLLA + PDLA) in the blend specimen; it equals 1.000 for neat PLLA or PDLA blends and falls below unity for PLLA–PEG–PLLA specimens, most significantly for PEG35k (*W*_PLA_ = 0.741). Δ*H*_c_ is subtracted from Δ*H*_m1_ in eq 1 because cold crystallization during the DSC heating ramp produces exclusively homocrystals, not SC crystals; Δ*H*_SC_ in eq 2 requires no such correction because SC crystals nucleate only from the melt during cooling.^7,28^

A mechanistic refinement applies to L100 K/PDLA and L200 K/PDLA, both of which display a shallow homocrystalline endotherm (*T*_m1_ ≈ 170–171 °C) alongside the dominant SC endotherm in the DSC heating scan. After *W*_PLA_ correction, Δ*H*_c_ exceeds Δ*H*_m1_, yielding χ_homo_ ≈ 0. This result confirms that the observed homocrystalline endotherm is a DSC heating-ramp artifact arising from cold crystallization; no homocrystals were present in the as-molded specimen. Therefore, all crystallization that occurred during mold cooling for these specimens was stereocomplexation, and *f*_SC_ = 100%. The SC formation efficiency was evaluated using

3 $$f_{SC}\;\left(\%\right)=\chi_{SC}/\left(\chi_{SC}+\chi_{homo}\right)\times 100$$

#### 2.5.4 X-ray Diffraction

Wide-angle X-ray diffraction patterns were collected on a Bruker D8 Advance diffractometer (Cu Kα radiation, λ = 1.5406 Å; 40 kV, 40 mA; scan range 2θ = 2–40°; step size 0.02°; scan rate 2° min^–1^). Reflection assignments follow established literature: α-form homocrystalline PLA at 2θ ≈ 14.3°, 16.3°, 18.6°, and 22.0°;^37,38^ SC triclinic crystals at 2θ ≈ 11.9°, 20.6°, and 23.8° corresponding to the (110), (300)/(030), and (220) planes.^33,34^ Quantitative phase fractions were determined by Gaussian peak deconvolution.

#### 2.5.5 Tensile Testing

Tensile properties were measured according to ASTM D638–10 (type I specimen geometry; gauge length 50 mm; crosshead speed 1 mm min^–1^; ambient conditions ≈ 23 °C, 50% RH) using a Narin Instrument NRI-TS500–2S universal testing machine. Five specimens per formulation (*n* = 5) were tested. Young’s modulus (*E*) was determined from the initial linear slope of the stress–strain curve. Toughness was estimated by the triangular approximation

7 $$toughness\;\left(MJ{\;m}^{-3}\right)=1/2\times \sigma_{b}\times \varepsilon_{b}$$

where σ_b_ is the tensile strength at break (MPa) and ε_b_ is the elongation at break.

#### 2.5.6 Heat-Resistance Evaluation

Injection-molded bars (dimensions: 13 × 125 × 4 mm) were placed horizontally in a forced-convection hot-air oven at 100 °C for 30 min, then transferred to 200 °C for a further 30 min. Dimensional stability and macroscopic morphological changes were documented photographically immediately after each exposure step.

## 3 Results and Discussion

### 3.1 Synthesis Optimization and Molecular Weight Characterization

The synthesis of PLLA and PLLA–PEG-PLLA triblock copolymers was optimized with respect to catalyst loading and reaction temperature (Table 2). Standard bulk ROP conditions (0.1 mol % Sn­(Oct)_2_, 160 °C, 4 h, nitrogen) gave ≥99% monomer conversion for PLLA homopolymers and PEG4k-initiated copolymers, with PDI = 1.12–1.26 for the higher-*M*_n_ series. PEG400 is a low-viscosity liquid that instantly miscibilizes with molten l-lactide, generating local exotherms; reducing the catalyst concentration 10-fold to 0.01 mol % suppressed volatility and maintained polymerization control. The resulting PDI values of 1.65 (L50K-400-L50K) and 1.10 (L100 K-400-L100 K) demonstrate that the broader dispersity is limited to the low-[L-LA]/[I] (700:1) synthesis, a known consequence of fast initiation kinetics at this ratio.^2^ PEG35k (*T*_m_ ≈ 65 °C) was polymerized at 140 °C; the reduced temperature ensures complete, uniform macroinitiator melting before chain growth begins and eliminates hot spots that cause discoloration at 160 °C. Under these tailored conditions, all PEG35k-initiated reactions reached 98% conversion with no visual signs of degradation, confirming adequate thermal stability of the high-*M*_n_ triblocks.

*M*_n_ values by GPC ranged from 48,000 to 180,000 g/mol (Table 2). PDI for the higher-*M*_n_ series (L200 K, L100 K-x-L100 K) fell within 1.10–1.26, confirming controlled ROP kinetics. GPC *M*_n_ values were lower than *M*_n,NMR_ for PEG35k-containing copolymers: for L50K-35k-L50K, *M*_n, NMR_ = 96,000 vs *M*_n, GPC_ = 75,200 g/mol (discrepancy 28%). This underestimation is a well-documented consequence of polystyrene-standard GPC calibration for PLA–PEG copolymers, whose compact coil dimensions in THF yield hydrodynamic volumes smaller than linear PS of equivalent molar mass;^36^ Xie et al.^29^ reported a comparable offset under identical GPC conditions. The NMR/GPC cross-validation therefore confirms compositional control even where absolute GPC values are underestimated. All polymers formed complete, self-supporting solution-cast films, confirming that molecular weight exceeded the critical entanglement threshold for cohesive film behavior. Below *M*_n_ ≈ 80,000 g/mol (GPC), injection-molded bars lacked dimensional stability, consistent with the minimum entanglement density required for melt-flow coherence in semicrystalline PLA.^33^ These findings underscore the importance of matching ROP conditions to the physical state of the macroinitiator.

### 3.2 Triblock Copolymer Architecture Confirmation by ^1^H NMR

Triblock copolymer architecture was confirmed for PLLA–PEG–PLLA compositions by ^1^H NMR (Figure 2). PLLA homopolymers show only the lactide methine quartet at δ 5.16 ppm (peak a, 1H per repeat unit) and the methyl doublet at δ 1.59 ppm (peak b, 3H).^34^ All triblock copolymers exhibit PLLA resonances with three additional signals diagnostic of covalent PEG incorporation: (i) the PEG −OCH_2_CH_2_O– methylene singlet at δ 3.65 ppm (peak c), whose intensity scales with PEG block length;^31^ (ii) the PEG–ester α-methylene multiplet at δ 4.25 ppm (peak d), which directly evidence covalent connectivity at the PLLA–PEG joint;^35^ and (iii) terminal lactyl methyl protons at δ 1.25 ppm (peak e) arising from the macroinitiator chain end. The c/a integral ratio provides an independent *M*_n,NMR_ estimate (Table 2) that cross-validates the GPC data.

**Figure 2:**
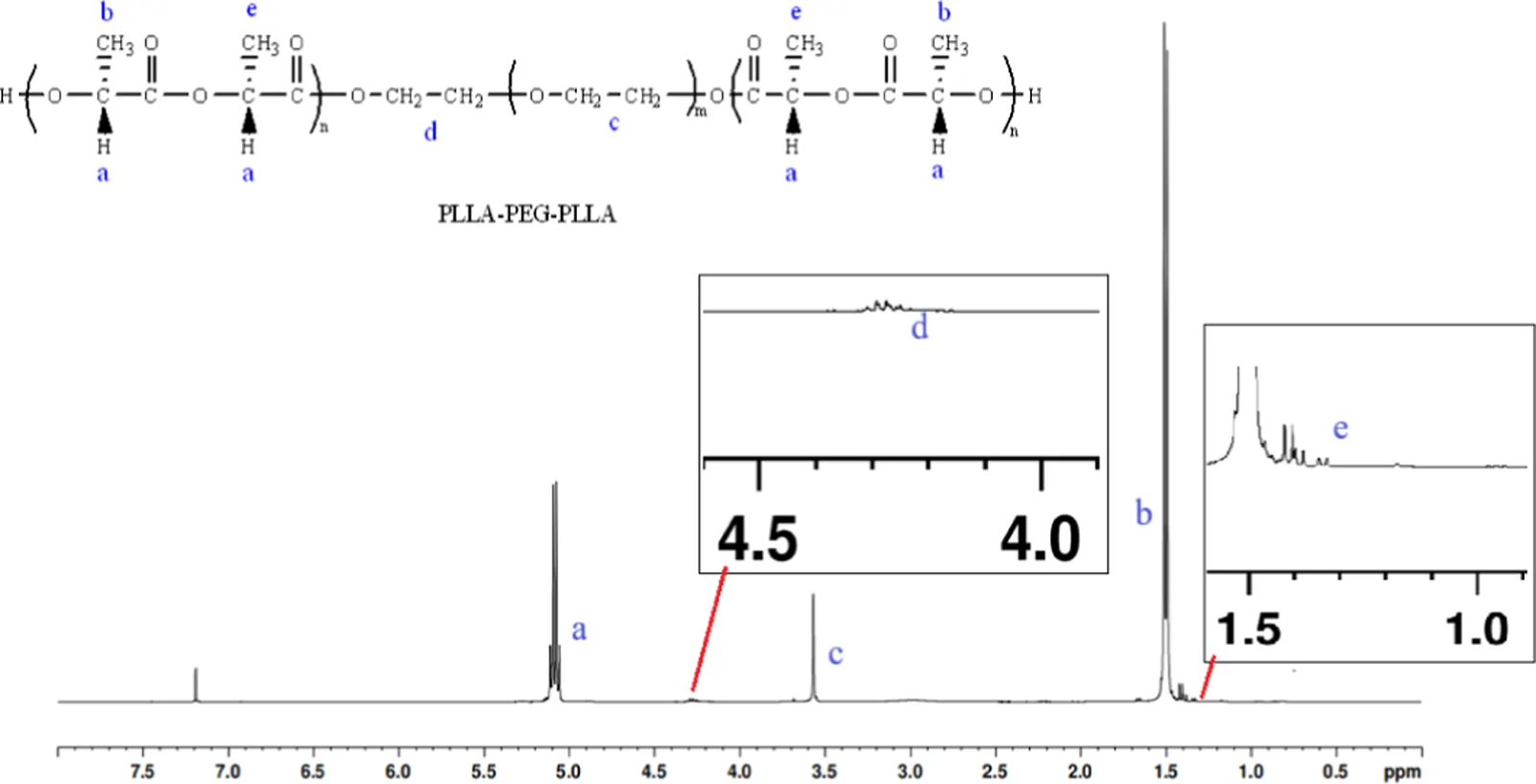
^1^H NMR spectra (400 MHz, CDCl_3_) of representative PLLA and PLLA–PEG–PLLA copolymers. Peak assignments: (a) PLLA methine, δ 5.16 ppm; (b) PLLA methyl, δ 1.59 ppm; (c) PEG methylene (−OCH_2_CH_2_O−), δ 3.65 ppm; (d) PEG–ester linkage α-methylene, δ 4.25 ppm; (e) terminal lactyl methyl, δ 1.25 ppm.

### 3.3 Thermal Analysis by DSC

DSC thermograms of all injection-molded specimens are shown in Figure 3. All χ_homo_ and χ_SC_ values are *W*_PLA_-corrected per eqs 1 and 2. Table 3 reports both *W*_PLA_-corrected and uncorrected (Δ*H*-based) crystallinity values to illustrate the magnitude of the correction. *W*_PLA_ correction is essential for high-PEG-content formulations. For L50K-35k-L50K (*W*_PLA_ = 0.741), the uncorrected χ_homo_ = 49.1% underestimates the true PLLA-fraction crystallinity by 17 percentage points (pp); the corrected value is 66.1% (Table 3). For PEG400 and PEG4k samples the correction is ≤2 pp because the PEG mass fraction is ≤4 wt %. The *f*_SC_ metric (eq 3) captures the competition between SC and homocrystallization in a single normalized number, enabling direct comparison across all formulations.

**Figure 3:**
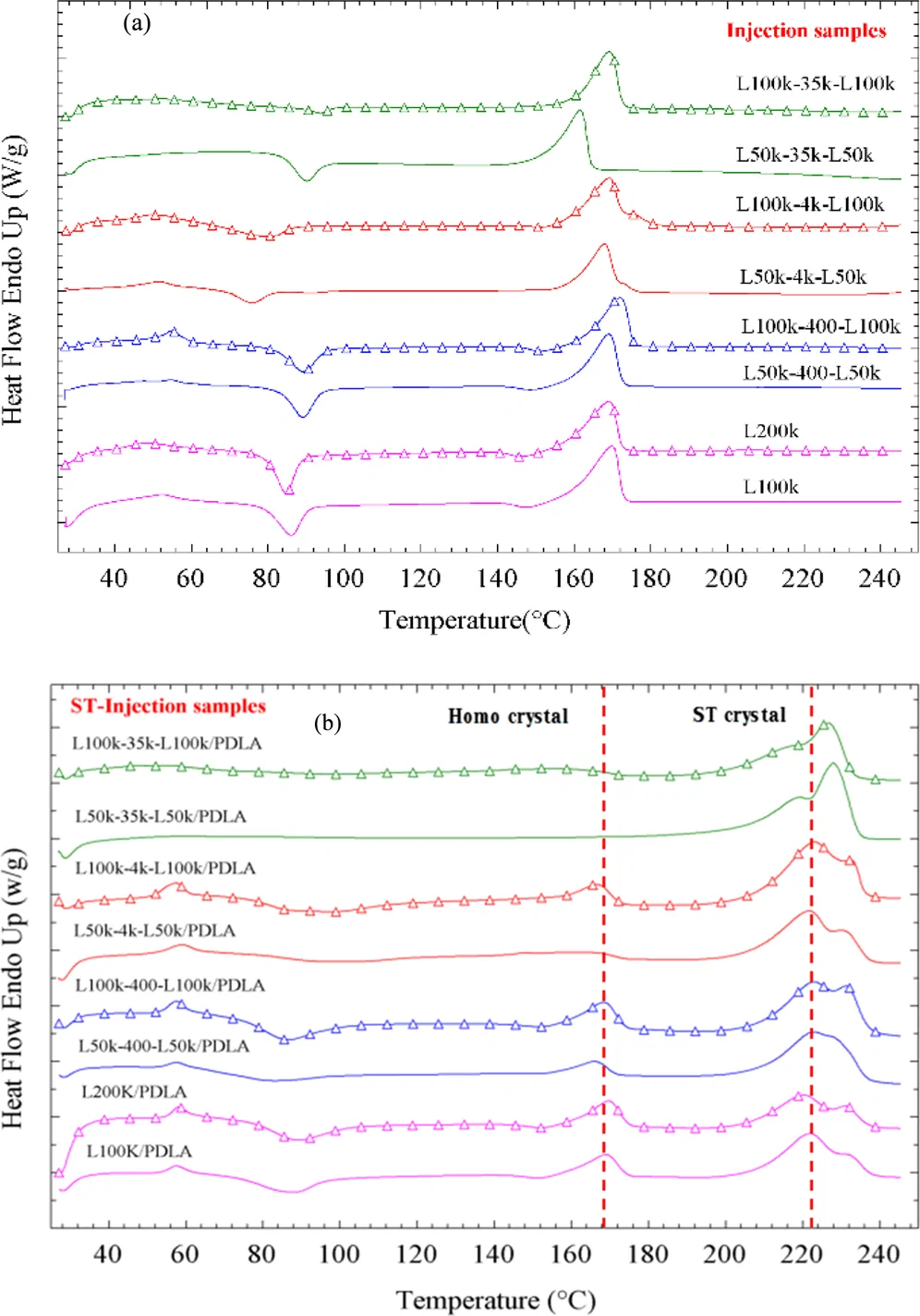
DSC thermograms (first heating scan, 10 °C min^–1^, N_2_) of injection-molded specimens: (a) non-SC PLLA and PLLA–PEG–PLLA samples; (b) SC-blend specimens.

**Table 3:** DSC Thermal Properties of Injection-Molded Specimens

sample	*T*_g_ (°C)	*T*_c_ (°C)	Δ*H*_c_ (J g^–1^)	*T*_m1_ (°C)	χ_homo,raw_ (%)	χ_homo,corr_ (%)	*T*_m2_ (°C)	χ_SC,raw_ (%)	χ_SC,corr_ (%)/*f*_SC_ (%)
L100 K	53	97	5.3	167	38.0	38.0	—	—	—/—
L200 K	55	99	3.1	172	32.1	32.1	—	—	—/—
L50K-400-L50K	46	80	4.1	164	46.6	47.2	—	—	—/—
L100 K-400-L100 K	47	84	3.4	169	44.0	44.5	—	—	—/—
L50K-4k-L50K	44	76	1.6	170	50.6	52.3	—	—	—/—
L100 K-4k-L100 K	45	79	1.9	171	48.5	49.5	—	—	—/—
L50K-35k-L50K	45	90	9.6	165	49.1^b^	66.1	—	—	—/—
L100 K-35k-L100 K	46	94	5.2	168	55.4^b^	61.3	—	—	—/—
L100 K/PDLA	54	91	4.2	170	0^a^	0^a^	222	30.2	30.2/100
L200 K/PDLA	56	93	3.8	171	0^a^	0^a^	220	25.1	25.1/100
L50K-400-L50K/PDLA	49	89	3.1	165	10.8	11.0	220	34.3	34.7/76.0
L100 K-400-L100 K/PDLA	50	87	2.8	167	13.1	13.3	220	41.8	42.5/76.2
L50K-4k-L50K/PDLA	47	82	1.4	—	3.1	3.2	225	42.8	43.7/93.2
L100 K-4k-L100 K/PDLA	48	85	1.2	—	2.2	2.3	224	40.2	41.0/94.7
L50K-35k-L50K/PDLA	45	—	—	—	0	0	227	41.4^b^	55.9/100
L100 K-35k-L100 K/PDLA	46	—	—	—	0	0	226	30.4^b^	41.0/100

a χ_homo_ ≈ 0 after *W*_PLA_ correction because Δ*H*_c_ ≥ Δ*H*_homo_; *f*_SC_ = 100% because all in-mold crystallization was stereocomplexation.

b *W*_PLA_ = 0.741 for all PEG35k specimens; raw χ_homo_ = 49.1% corrected to 66.1% (+17 pp) for non-SC PEG35k; same *W*_PLA_ applies to SC counterparts.

All non-SC specimens showed homocrystalline melting endotherms at 161–172 °C. *T*_g_ of neat PLLA was 53 °C (L100 K) and 55 °C (L200 K). PEG incorporation progressively reduced *T*_g_ by up to 8–9 °C for PEG35k copolymers (45–46 °C), consistent with Bikiaris et al.^26^ The *W*_PLA_-corrected χ_homo_ increased from 38.0% (L100 K) to 66.1% (L50K-35k-L50K), a 28-pp enhancement. Comparison with solution-cast films (Table S1, Supporting Information) reveals a processing-induced crystallinity deficit of approximately 7 pp for PEG35k and approximately 12 pp for neat PLLA, arising from the rapid quench of injection molding.

SC specimens showed characteristic SC melting endotherms at 220–227 °C. PEG400 SC specimens retained visible homocrystalline endotherms, yielding *f*_SC_ ≈ 75–76%. PEG4k SC specimens showed near-complete SC formation (*f*_SC_ = 92.9–94.7%), but corresponding solution-cast SC films showed complete SC formation (*f*_SC_ = 100%, Table S1 in Supporting Information), demonstrating that PEG4k provides sufficient chain mobility for complete stereocomplexation under slow evaporation but falls short under rapid injection-mold cooling. PEG35k SC specimens exhibited single endotherms at 226–227 °C with no homocrystalline peak (χ_SC_ = 55.9%, *f*_SC_ = 100%), establishing *M*_n,PEG_ ≥ 35,000 g/mol.

For L100 K/PDLA and L200 K/PDLA, dual endotherms at approximately 170 °C and 220–222 °C were observed. As detailed in Section 2.5.3, the shallow homocrystalline endotherm (*T*_m1_ ≈ 170–171 °C) in these specimens is a DSC heating-ramp artifact: after *W*_PLA_ correction, Δ*H*_c_ exceeds Δ*H*_m1_, yielding χ_homo_ ≈ 0. The arithmetic is explicit: for L100 K/PDLA, Δ*H*_c_ = 4.2 J g^–1^ and Δ*H*_m1_ = 4.2 J g^–1^ (apparent), so (Δ*H*_m1_ – Δ*H*_c_) ≈ 0 before correction; *W*_PLA_ = 1.000 for the neat-PLLA blend, so χ_homo_ = 0/(93 × 1.000) = 0. This confirms that no homocrystals were present in the as-molded specimen, all crystallization occurring during mold cooling was stereocomplexation, and *f*_SC_ = 100% for both neat-PLLA SC specimens. As shown in Table 4, XRD reveals residual homocrystalline α-form peaks for L100 K/PDLA and L200 K/PDLA.

**Table 4:** Relative Phase Fractions From XRD Gaussian Peak Deconvolution

sample	*X*_HC_ (%)	*X*_SC_ (%)	*X*_am_ (%)	*f*_SC,XRD_ (%)
L100 K	29.1	—	70.9	—
L200 K	24.8	—	75.2	—
L50K-400-L50K	27.3	—	72.7	—
L100 K-400-L100 K	28.6	—	71.4	—
L50K-4k-L50K	34.2	—	65.8	—
L100 K-4k-L100 K	32.7	—	67.3	—
L50K-35k-L50K	47.8	—	52.2	—
L100 K-35k-L100 K	43.5	—	56.5	—
L100 K/PDLA	12.4	22.3	65.3	64.3
L200 K/PDLA	10.6	18.7	70.7	63.8
L50K-400-L50K/PDLA	9.1	27.2	63.7	74.9
L100 K-400-L100 K/PDLA	8.3	25.8	65.9	75.7
L50K-4k-L50K/PDLA	2.8	31.6	65.6	91.8
L100 K-4k-L100 K/PDLA	2.1	30.4	67.5	93.5
L50K-35k-L50K/PDLA	—	40.2	59.8	100.0
L100 K-35k-L100 K/PDLA	—	30.5	69.5	100.0

The absence of cold-crystallization exotherms in PEG35k SC specimens confirms that complete SC crystallization occurred during mold cooling without additional thermal input. For non-SC specimens, PEG4k copolymers showed the lowest *T*_c_ (76–79 °C) and Δ*H*_c_, indicating the fastest crystallization kinetics. PEG35k copolymers showed slightly higher *T*_c_ (90–94 °C) but notably lower Δ*H*_c_ (5.2–9.6 J g^–1^), confirming that most crystallization occurred during cooling. In SC specimens, PEG35k entirely eliminated cold crystallization. This contrast between PEG4k (*f*_SC_ < 100% in injection-molded specimens) and PEG35k (*f*_SC_ = 100%) defines a practical design window: *M*_n,PEG_ ≥ 35,000 g/mol is required to achieve complete in-mold SC formation in *M*_n_ ≈ 200,000 g/mol blends without postmold annealing.

The monotonic increase in fSC with PEG block molecular weight reflects three cooperative mechanisms. First, longer PEG chains are more effective plasticizers of the PLLA matrix: higher PEG Mn reduces the local glass-transition temperature of the interphase region around each PEG domain, decreasing segmental friction and increasing diffusivity of PLLA and PDLA chain segments during mold cooling - enhanced mobility directly accelerates the enantiomeric chain-recognition step required for SC lattice nucleation.^23–25^ Second, PEG35k chains are long enough to form microphase-separated soft-segment domains that act as heterogeneous nucleation sites for SC crystallites, analogous to the role of selective nucleating agents in promoting SC formation in high-*M*_w_ blends.^18^ Third, longer PEG blocks extend further into the surrounding PLLA matrix, releasing chain-end mobility over a larger cooperative volume and allowing more PLLA–PDLA pairs to achieve the spatial register required for SC cocrystallization within the rapid mold-cooling window. The progressive nature of this enhancement (fSC: 75–76% for PEG400; 93–95% for PEG4k; 100% for PEG35k) suggests that all three contributions scale with PEG chain length in this molecular weight range.

### 3.4 XRD Crystalline Structure

XRD patterns (Figure 4) confirmed the DSC trends. Neat PLLA and PEG400 specimens showed predominantly amorphous halos. PEG4k and PEG35k samples displayed well-resolved α-form peaks at 2θ = 14.3°, 16.3°, 18.6°, 22.0°.^37,38^ All SC specimens showed characteristic SC triclinic peaks at 2θ = 11.9°, 20.6°, 23.8° [(110), (300)/(030), (220) planes].^37,38^ PEG35k SC showed SC peaks with complete suppression of α-form reflections.

**Figure 4:**
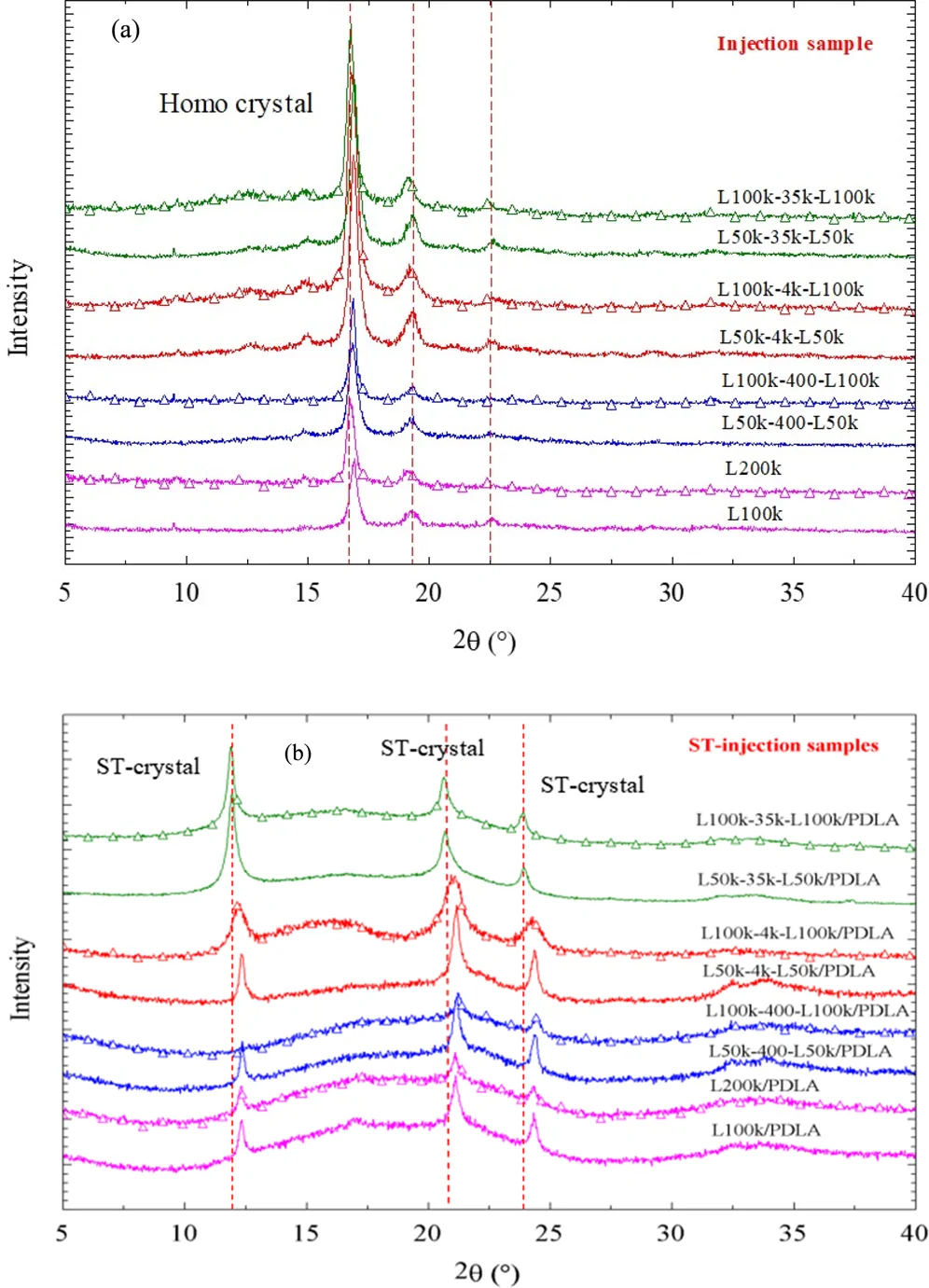
XRD patterns (Cu Kα, λ = 1.5406 Å; 2θ = 2–40°) of samples. (a) Non-SC PLLA and PLLA–PEG–PLLA samples; (b) SC-blend specimens.

Three contributions were resolved by Gaussian fitting following Xie et al.^29^ homocrystalline (*A*_HC_, centered near 16.3°), SC crystalline (*A*_SC_, near 20.6°), and amorphous halo (*A*_am_)­

4 $$X_{HC}\;\left(\%\right)=A_{HC}/\left(A_{HC}+A_{SC}+A_{am}\right)\times 100$$

5 $$X_{SC}\;\left(\%\right)=A_{SC}/\left(A_{HC}+A_{SC}+A_{am}\right)\times 100$$

6 $$f_{SC,XRD}\;\left(\%\right)=A_{SC}/\left(A_{HC}+A_{SC}\right)\times 100$$

Results are compiled in Table 4. A linear regression of χ_SC_(DSC) vs *X*_SC_(XRD) across the SC samples yielded *R* ^2^ = 0.93, confirming the mutual consistency and robustness of the two measurement approaches. The *f*_SC,XRD_ values mirror DSC-derived *f*_SC_ values throughout: PEG400 SC gave *f*_SC,XRD_ ≈ 75%, PEG4k SC ≈ 92–94%, and PEG35k SC 100.0%, with no detectable homocrystalline diffraction.

### 3.5 Mechanical Properties

Tensile properties and toughness are summarized in Table 5 and shown in Figures 5 and 6. PEG incorporation produced the classical plasticizer-driven inverse balance between strength and ductility across the 100 K series: tensile strength decreased monotonically from 45 ± 4.7 MPa (L100 K) to 27 ± 4.4 MPa (L100 K-35k-L100 K), while elongation at break increased 8-fold from 45 ± 2.7% to 356 ± 3.6%. This trend is mechanistically consistent with PEG-induced reduction of intermolecular cohesion and increase in free volume, both of which facilitate chain-segment displacement under load.^23–25^ The higher elongation in injection-molded specimens compared to solution-cast films (e.g., 356% vs 87 ± 4.5% for L100 K-35k-L100 K; Table S1) reflects the larger amorphous fraction retained by rapid quench cooling, providing a larger reservoir of deformable, noncrystalline material.

**Table 5:** Tensile Properties and Toughness of Injection-Molded Specimens

sample	E (GPa)	tensile strength (MPa)	elongation at break (%)	toughness (MJ m–3)
L100 K	3.2 ± 0.2	45 ± 4.7	45 ± 2.7	10.1
L200 K	2.9 ± 0.3	33 ± 3.4	41 ± 3.1	6.8
L50K-400-L50K	2.5 ± 0.2	30 ± 3.2	48 ± 3.8	7.2
L100 K-400-L100 K	2.8 ± 0.3	35 ± 4.1	52 ± 4.2	9.1
L50K-4k-L50K	1.8 ± 0.2	25 ± 3.8	188 ± 5.1	23.5
L100 K-4k-L100 K	2.1 ± 0.2	30 ± 4.3	236 ± 4.9	35.4
L50K-35k-L50K	1.2 ± 0.1	22 ± 3.6	298 ± 4.8	32.8
L100 K-35k-L100 K	1.5 ± 0.2	27 ± 4.4	356 ± 3.6	48.1
L100 K/PDLA	3.5 ± 0.3	40 ± 3.5	78 ± 4.2	15.6
L200 K/PDLA	3.1 ± 0.2	36 ± 4.1	82 ± 3.9	14.8
L50K-400-L50K/PDLA	3.3 ± 0.2	42 ± 3.8	110 ± 5.2	23.1
L100 K-400-L100 K/PDLA	3.6 ± 0.3	45 ± 4.2	124 ± 4.8	27.9
L50K-4k-L50K/PDLA	3.8 ± 0.2	46 ± 4.0	188 ± 5.6	43.2
L100 K-4k-L100 K/PDLA	4.0 ± 0.3	48 ± 3.6	218 ± 5.1	52.3
L50K-35k-L50K/PDLA	3.9 ± 0.2	50 ± 4.5	248 ± 4.6	62.0
L100 K-35k-L100 K/PDLA	4.2 ± 0.3	53 ± 4.2	270 ± 3.8	71.5

**Figure 5:**
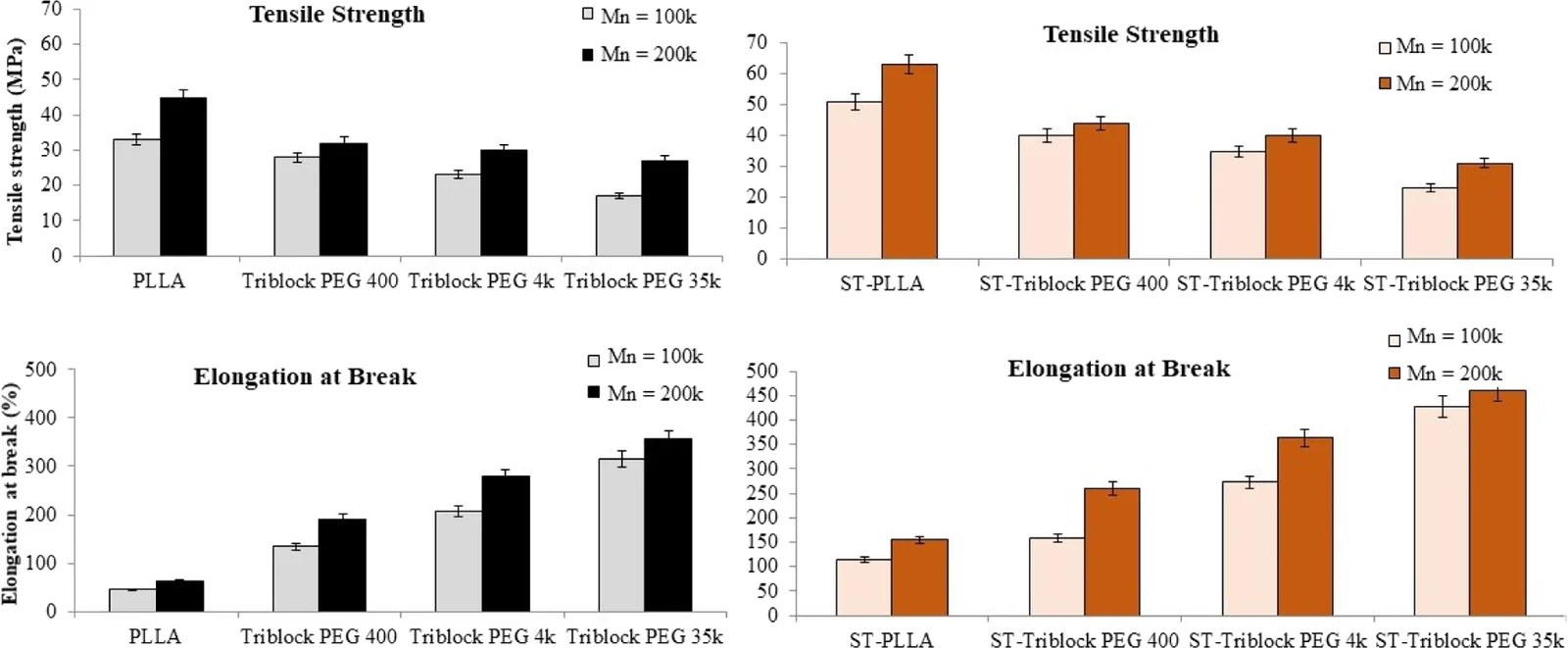
Tensile strength and elongation at break of injection-molded non-SC and SC specimens with different PEG middle-block molecular weights and PLLA block molecular weights.

**Figure 6:**
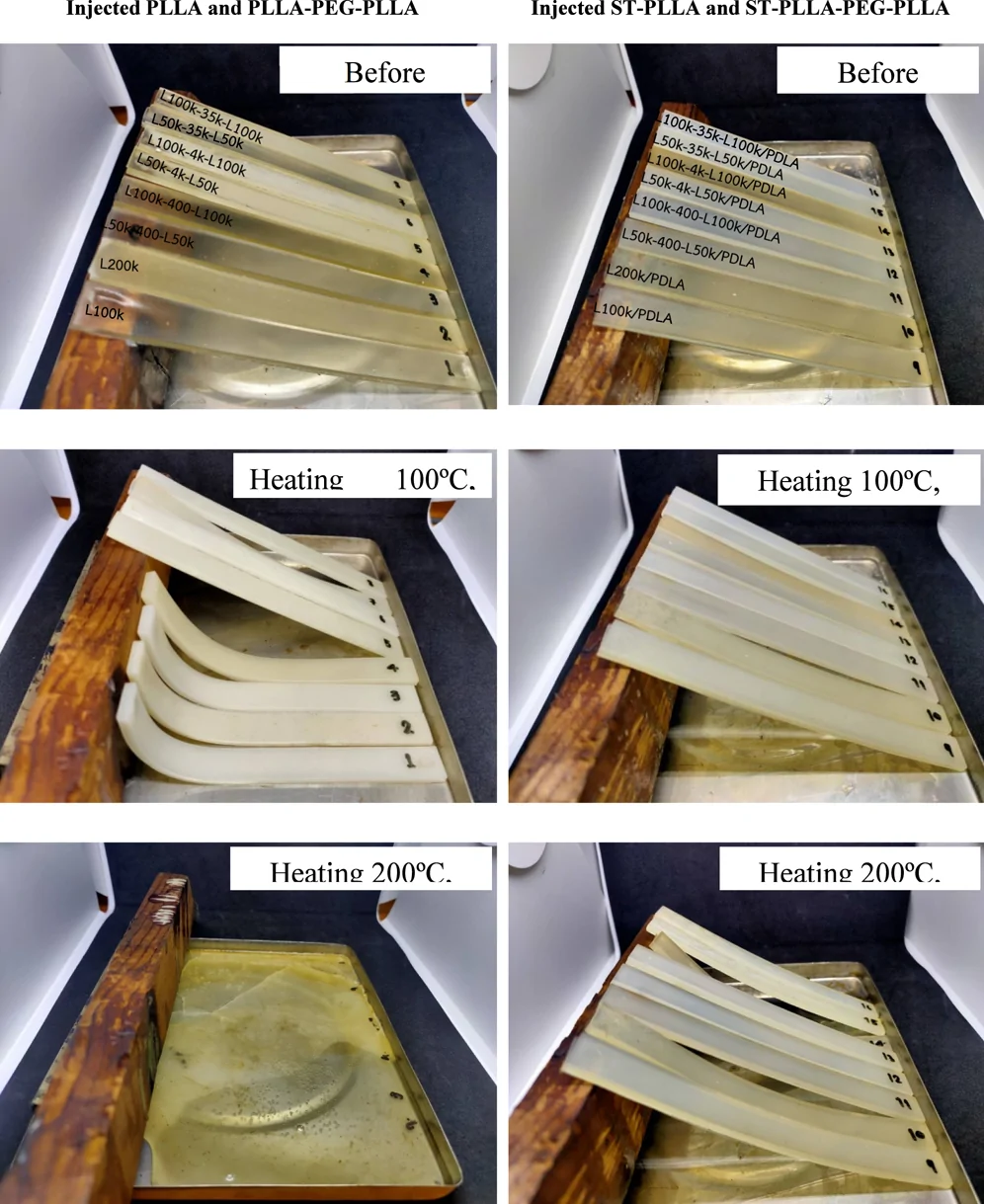
Heat-resistance photographs of injection-molded non-SC and SC specimens (13 × 125 × 4 mm) before heating and after sequential oven exposure at 100 °C for 30 min and 200 °C for 30 min.

Stereocomplex formation conferred qualitatively different behavior: both strength and toughness improved relative to the corresponding non-SC specimen, despite the 1:1 mass dilution with PDLA. For example, L100 K/PDLA (SC) showed 40 ± 3.5 MPa and 78 ± 4.2% elongation, compared with L100 K: 45 ± 4.7 MPa and 45 ± 2.7%. The reduction in peak strength is offset by a 73% increase in elongation, yielding a 1.54-fold improvement in toughness from 10.1 to 15.6 MJ m^–3^. The mechanism is well established: SC crystallites distributed throughout the amorphous matrix act as dense physical cross-links, suppressing brittle fracture initiation and propagation through load redistribution across the covalently bonded network.^8,28^ The SC lattice is uniquely effective as a physical cross-link because of the high density of C–H···O=C hydrogen bonds within the triclinic unit cell.^8^

For non-SC specimens, toughness increased from 10.1 MJ m^–3^ to 48.1 MJ m^–3^, a 4.8-fold improvement. For SC specimens, the maximum rose from 28.8 MJ m^–3^ to 71.5 MJ m^–3^, representing further 1.49-fold enhancement (71.5 vs 48.1 MJ m-^3^) and a 7.1-fold improvement relative to neat L100 K. The synergy arises from the dense C–H···O=C hydrogen-bond network in the SC lattice providing physical cross-linking points for load transfer while PEG segments maintain chain mobility for plastic deformation.^8,28^

Within each PEG grade, specimens with higher PLLA block consistently showed higher strength and elongation than the L50K series, attributed to greater chain entanglement density.^29^ An exception is neat PLLA: L100 K (45 ± 4.7 MPa) outperformed L200 K (33 ± 3.4 MPa) because of its higher crystallinity (38.0% vs 32.1%), as crystallinity strongly governs the tensile strength of semicrystalline PLA. It is acknowledged that higher Mn (L200 K) should, in principle, increase tensile strength through greater chain entanglement density. However, for rapidly quench-cooled injection-molded specimens, the crystallite network is the dominant load-bearing element and the lower crystallinity of L200 K (32.1%) substantially reduces this reinforcement. The entanglement contribution cannot compensate for reduced crystallite density in amorphous-rich specimens produced under rapid cooling conditions.^39^

### 3.6 Heat Resistance

Results of the sequential 100 °C/200 °C heat-resistance test are shown in Figure 6. Non-SC specimens showed crystallinity-dependent behavior: PEG400-containing copolymers deformed severely at 100 °C because amorphous regions above *T*_g_ (approximately 45–55 °C) softened without sufficient crystalline reinforcement; PEG4k and PEG35k copolymers retained shape at 100 °C but melted completely at 200 °C, consistent with *T*_m_ = 161–172 °C.

All SC specimens maintained dimensional stability at 100 °C. At 200 °C, L100 K/PDLA and L200 K/PDLA showed slight bending from melting of their residual homocrystalline fraction while the SC network (*T*_m_ = 220–222 °C) remained intact. All SC–PLLA–PEG–PLLA formulations maintained full dimensional stability at 200 °C (approximately 20–27 °C below *T*_m2_ = 220–227 °C), confirming that *f*_SC_ ≥ 92.9% is sufficient to preserve structural integrity at near-melt temperatures. PEG35k SC specimens (*f*_SC_ = 100%, *T*_m2_ = 226–227 °C) showed zero deformation.

## 4 Conclusions

This study investigated the influence of PEG middle-block molecular weight on SC crystallization, thermal properties, mechanical performance, and heat resistance of high-*M*_n_ injection-molded PLLA–PEG–PLLA/PDLA blends. A minimum *M*_n_ ≈ 80,000 g/mol is required for coherent injection-molded specimens. Solution-cast film formation provides a rapid screening test for molecular weight adequacy before melt processing. All polymers were synthesized close to target *M*_n_ (PDI = 1.10–1.65; narrow PDI = 1.10–1.26 for the higher-*M*_n_ series). Macroinitiator-specific optimization of catalyst loading and temperature was essential. *W*_PLA_ correction revealed a 28 pp increase in homocrystallinity (38.0% to 66.1% for PEG35k), more than double the apparent increase from uncorrected values. *f*_SC_ increased monotonically with PEG molecular weight, reaching 100% for PEG35k, independently confirmed by XRD. Solution-cast SC films showed *f*_SC_ = 100% for PEG4k but injection-molded counterparts gave *f*_SC_ = 92.9–94.7%, quantifying the kinetic barrier of rapid mold cooling. PEG35k overcomes this barrier, achieving *f*_SC_ = 100% under both processing modes. PEG35k SC specimens crystallized completely during mold cooling without cold crystallization. PEG alone increased toughness 4.8-fold; SC formation added a further 1.5-fold boost, yielding a maximum of 71.5 MJ m^–3^ and a combined 7.1-fold improvement over neat PLLA. All SC–PLLA–PEG–PLLA specimens maintained full dimensional stability at 200 °C (within approximately 27 °C of *T*_m2_ = 220–227 °C), in contrast to non-SC counterparts that melted completely. *W*_PLA_-corrected χ_SC_, *f*_SC_, and XRD deconvolution together constitute a self-consistent analytical framework for cross-study comparison of SC-PLA systems. These findings provide quantitative design guidelines for developing high-performance, melt-processable SC-PLA materials suitable for injection molding. Future work incorporating rheological analysis would be valuable for directly elucidating the influence of PEG middle-block molecular weight on chain mobility and melt dynamics.

## Supplementary Material



## Notes

Synthesis procedures and reaction conditions; ^1^H NMR spectral analysis and chemical shift assignments; ^1^H NMR spectra of synthesized polymers; solution-cast film reference DSC data; and supplementary ROP conditions (PDF)
