# Supplementary material for: PEG Middle-Block Molecular Weight as a Quantitative Design Variable for Stereocomplex Formation Efficiency in High-Molecular-Weight PLLA–PEG–PLLA/PDLA Blends
Source: ACS Omega. 2026 Sep 25;11(39):60350–9. doi: 10.1021/acsomega.6c08171 (PMC13647841; doi:10.1021/acsomega.6c08171)
Supplement: Supplementary file 1 [file ao6c08171_si_001.pdf]

Supplementary Information

PEG Middle-Block Molecular Weight as a Quantitative Design Variable for Stereocomplex Formation Efficiency in High-Molecular-Weight PLLA-PEG-PLLA/PDLA Blends

Dutchanee Pholharn[1], Samaneh Dehghani[2], Onpreeya Veang-in[3], Yottha Srithep[4], *

[1] Department of Chemistry, Faculty of Science and Technology, Rajabhat Mahasarakham University, Mahasarakham 44000, Thailand
[2] Department of Wind Energy, Kunsan National University, Gunsan 54150, Republic of Korea
[3] Department of Mechanical Engineering, Faculty of Industry and Technology, Rajamangala University of Technology Isan, Sakon Nakhon 47160, Thailand
[4] Manufacturing and Materials Research Unit, Faculty of Engineering, Mahasarakham University, Mahasarakham 44150, Thailand
* Corresponding author. E-mail: yottha.s@msu.ac.th; Tel.: +66 910614566

Contents: Section S1: Synthesis Procedures and Reaction Conditions; Section S2: ¹H-NMR Spectral Analysis and Chemical Shift Assignments; Figures S1–S8: ¹H-NMR Spectra; Section S3: Solution-Cast Film Reference DSC Data – Table S1; Table S2: Supplementary ROP Conditions Detail.

Section S1: Synthesis of PLLA and PLLA–PEG–PLLA Triblock Copolymers

S1.1. Materials and Reagents

L-Lactide (purity ≥ 99.5%, Total Corbion PLA Ltd.) was purified by double recrystallisation from ethyl acetate at 70–80°C and dried under vacuum at 50°C for ≥ 24 h immediately prior to polymerisation. PDLA (grade L120, M_n = 90,000 g/mol, M_w = 152,000 g/mol) was obtained from Total Corbion PLA Ltd. Polyethylene glycol (PEG, three grades: 400, 4000, 35,000 g/mol), 1-dodecanol (98%), stannous 2-ethylhexanoate ($Sn(Oct)_2$, 92.5–95%), and ethyl acetate (99.5%) were purchased from Sigma-Aldrich and used as received.

S1.2. Ring-Opening Polymerisation (ROP) Procedure

PLLA homopolymers (L100K, L200K) and PLLA–PEG–PLLA triblock copolymers (six compositions) were synthesised by bulk ROP of L-lactide catalysed by $Sn(Oct)_2$. 1-Dodecanol served as the initiator for PLLA homopolymers; dihydroxyl-terminated PEG served as the bifunctional macroinitiator for triblock synthesis. Sample codes, [L-LA]/[I] ratios, and SC blend partners are given in Table 1 of the main manuscript. Detailed ROP conditions and molecular weight results are in Table 2 of the main manuscript, and are also reproduced in Table S2 of this document for convenience.

General procedure: Purified L-lactide (500 g per batch) was charged into a sealed, oven-dried glass reactor with the initiator and a weighed amount of $Sn(Oct)_2$ dissolved in minimal anhydrous toluene, under nitrogen purge. The reactor was heated to the target temperature with continuous stirring. After 4 h, a small aliquot (~10 mg) was withdrawn and assessed for monomer conversion by ^{1}H-NMR (>98% conversion confirmed in all cases). The product was dissolved in chloroform, precipitated into cold methanol (10× volume), filtered, and dried under vacuum at 40°C for 48 h. Polymers were stored under nitrogen at −20°C.

S1.3. Macroinitiator-Specific Optimisation

PEG400 (liquid, *T*m ≈ −4 to −8°C): High fluidity and complete miscibility with molten L-lactide at 160°C cause rapid simultaneous initiation and exothermic runaway at standard catalyst loading. Catalyst was reduced to 0.01 mol% to maintain control. PDI = 1.65 (L50K-400-L50K) and 1.10 (L100K-400-L100K), showing that the effect is stronger at lower [L-LA]/[I] ratios (700:1) than at 1400:1.

PEG35k (solid, *T*m ≈ 60–65°C): At 160°C, inconsistent melting of the large solid pellets creates local hot spots and thermal degradation (observed as yellowing). Reducing temperature to 140°C (still well above T_m of PEG35k) ensures uniform melt initiation before polymerisation begins. All PEG35k-initiated reactions achieved 98% conversion without discolouration.

PEG4k (solid, *T*m ≈ 54–58°C): Standard conditions (0.1 mol% $Sn(Oct)_2$, 160°C) were used without modification, yielding 99% conversion and PDI = 1.15 (L100K-4k-L100K) to 1.40 (L50K-4k-L50K).

Table S2. *Optimised ROP conditions for all synthesised polymers*

Sample	Init. State	Temp (°C)	Time (h)	$Sn(Oct)_2$ (mol%)	Conv. (%)	Mn,GPC (g/mol)	Mw,GPC (g/mol)	PDI	Mn,NMR (g/mol)	Film
L100K	Solid	160	4	0.1	99	53,700	90,100	1.60[a]	—	
L200K	Solid	160	4	0.1	99	160,000	180,000	1.12	—	
L50K-400-L50K	Liquid	160	4	0.01	99	60,600	100,121	1.65[a]	87,000	
L100K-400-L100K	Liquid	160	4	0.01	99	178,000	190,200	1.10	160,000	
L50K-4k-L50K	Solid	160	4	0.1	99	48,000	67,000	1.40[a]	74,000	
L100K-4k-L100K	Solid	160	4	0.1	99	170,000	195,000	1.15	174,000	
L50K-35k-L50K	Solid	140	4	0.1	98	75,200	95,000	1.26	96,000	
L100K-35k-L100K	Solid	140	4	0.1	98	180,000	196,000	1.10	205,000	

[a] *PDI > 1.30 for lower-Mn series attributed to broader initiation kinetics at 700:1 [L-LA]/[I]; all samples satisfied Mn ≥ 80,000 g/mol.*

Section S2: ¹H-NMR Spectral Analysis

S2.1. Peak Assignments and Molecular Weight Estimation

All polymers were characterised by ¹H-NMR at 400 MHz in $CDCl_3$ (Varian). Five characteristic resonances were used (Figure 2 of the main manuscript illustrates representative spectra):

Peak a: PLLA methine (–CH–) at δ = 5.16 ppm (quartet, 1H per lactide repeat). Diagnostic for the PLA backbone; present in all samples.

Peak b: PLLA methyl ($–CH_3$) at δ = 1.59 ppm (doublet, 3H per lactide repeat). Confirms stereoregular PLLA structure [30, 31].

Peak c: PEG methylene ($–OCH_2CH_2O–$) at δ = 3.65 ppm (singlet, 4H per ethylene oxide unit). Absent in PLLA homopolymers; present only in triblock copolymers [31].

Peak d: PEG–ester linkage α-methylene ($–COO–CH_2–$) at δ = 4.25 ppm (multiplet, 2H). Confirms covalent connectivity between the PLLA blocks and the PEG middle block [31].

Peak e: Terminal lactyl methyl at δ = 1.25 ppm (doublet). Arises from the chain-end lactyl unit adjacent to the initiator OH; used for Mn,NMR end-group calculation.

For PLLA–PEG–PLLA triblocks, $M_{n,NMR}$ was estimated from the c/a integral ratio:

$M_{n,PLLA} = (I_a / I_c) \times (M_{n,PEG} / 44) \times 144$ (S1)

where I_a is the integral of peak a, I_c is the integral of peak c, 44 g/mol = MW of the ethylene oxide repeat unit, $M_{n,PEG}$ is the nominal PEG macroinitiator MW (400, 4000, or 35,000 g/mol), and 144 g/mol = MW of the L-lactide repeat unit. The ratio I_a/I_c gives the mole ratio of lactide repeat units to ethylene oxide repeat units; multiplying by the number of ethylene oxide units per PEG chain ($M_{n,PEG}/44$) and by 144 g/mol converts this ratio into the total PLLA mass (both arms combined) attached per PEG macroinitiator chain, i.e. $M_{n,PLLA}$, total. Resulting $M_{n,NMR}$ values are reported in Table 2 of the main manuscript (also Table S2 above).

S2.2. Individual Sample NMR Analyses (Figures S1–S8)

S2.2.1. L100K

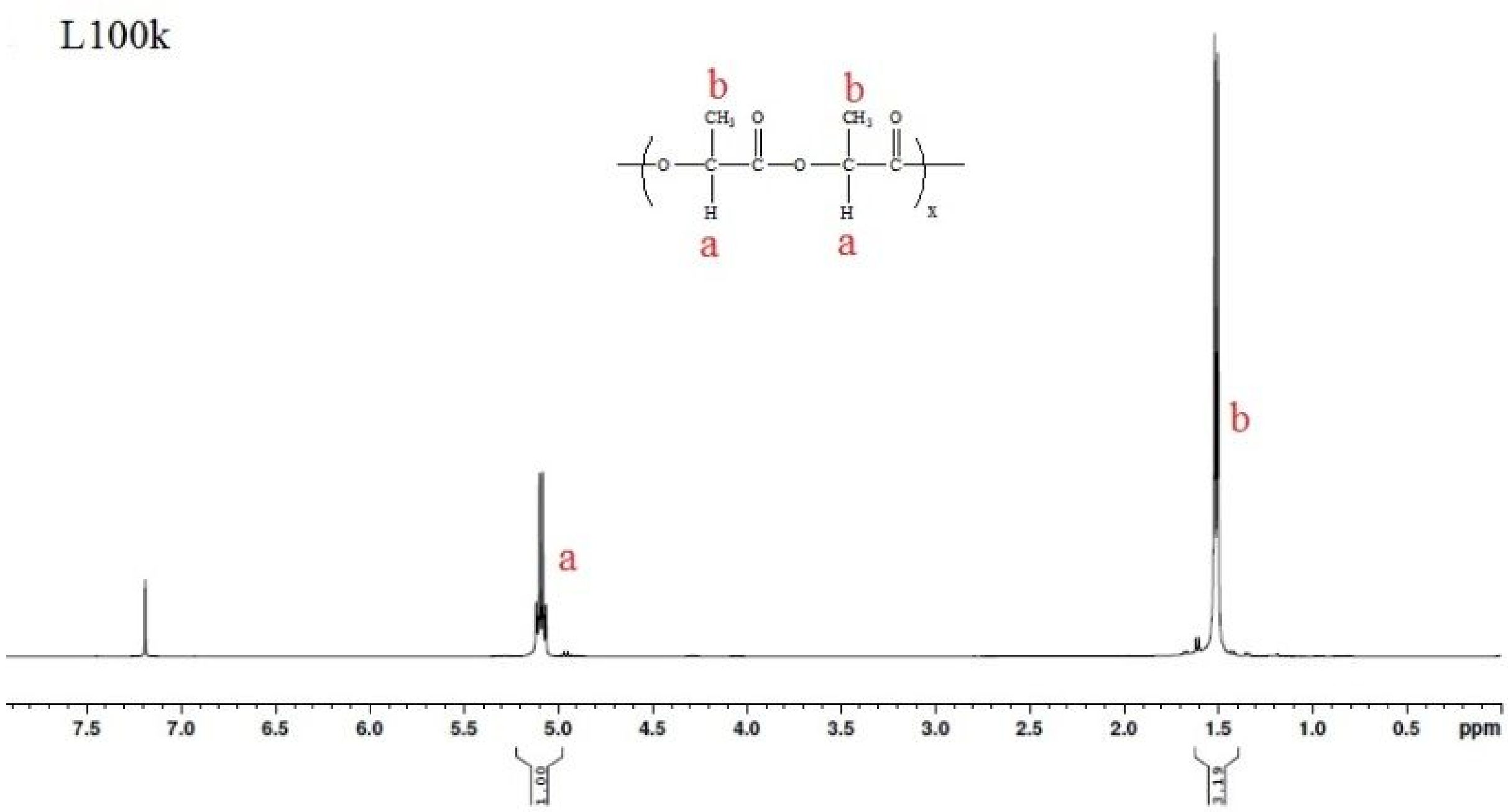

Figure S1. *¹H-NMR spectrum of L100K (PLLA homopolymer, target Mn = 100,000 g/mol) in $CDCl_3$ at 400 MHz.*

Spectrum shows peaks a (δ 5.16 ppm) and b (δ 1.59 ppm) in a 1:3 integral ratio, consistent with the PLLA repeat unit. No PEG signals (peak c) are observed, confirming homopolymer identity. Terminal methyl (peak e, δ 1.25 ppm) from 1-dodecanol chain end is visible as a minor doublet. Mn,GPC = 53,700 g/mol (PDI = 1.60); Mn,NMR not applicable (no PEG end-group).

S2.2.2. L200K

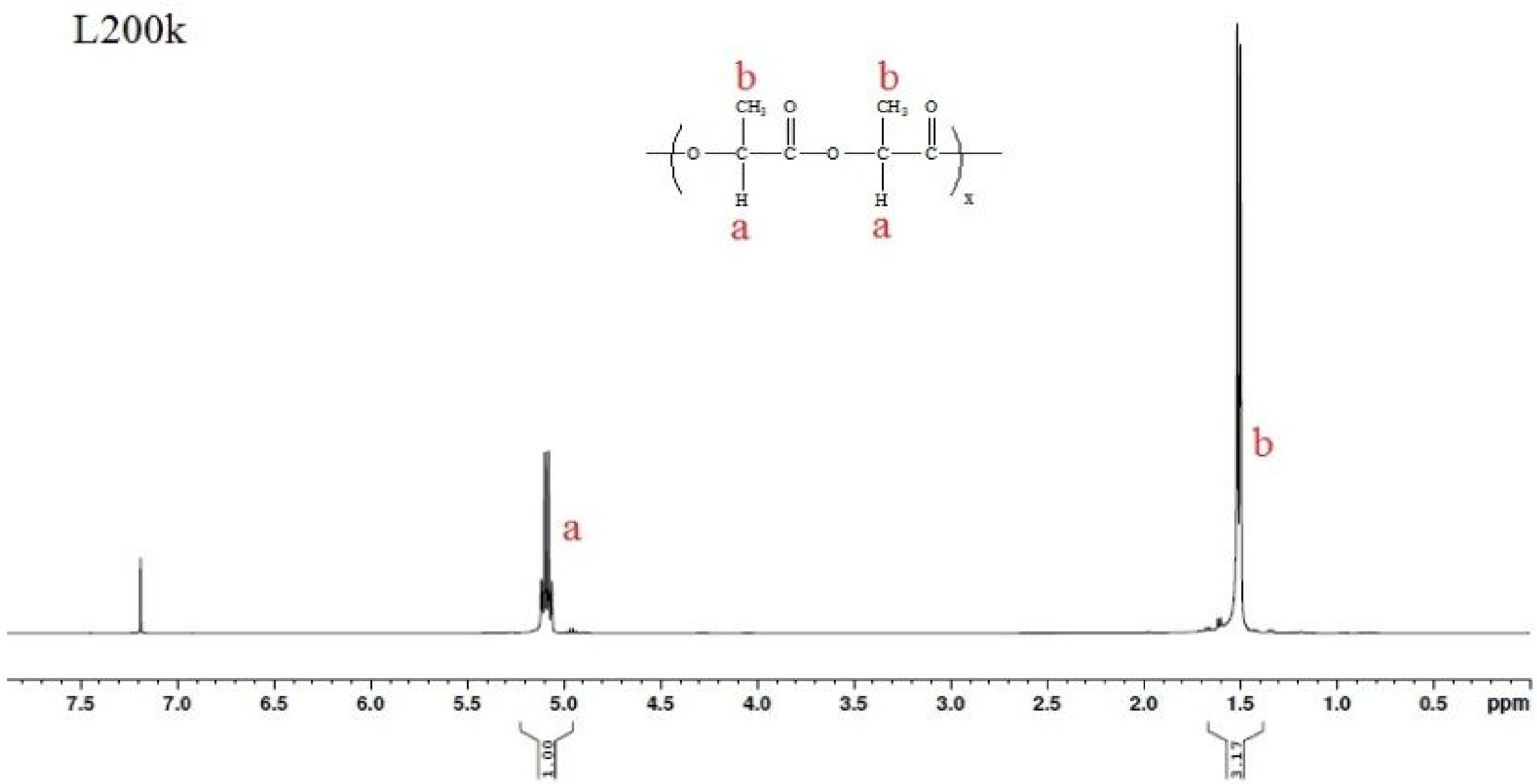

Figure S2. *^{1}H-NMR spectrum of L200K (PLLA homopolymer, target Mn = 200,000 g/mol) in $CDCl_3$ at 400 MHz.*

Identical peak pattern to L100K; peak e integrates to a smaller relative value, consistent with the higher molecular weight (fewer chain ends per unit mass). No PEG signals. Mn,GPC = 160,000 g/mol (PDI = 1.12).

S2.2.3. L50K-400-L50K

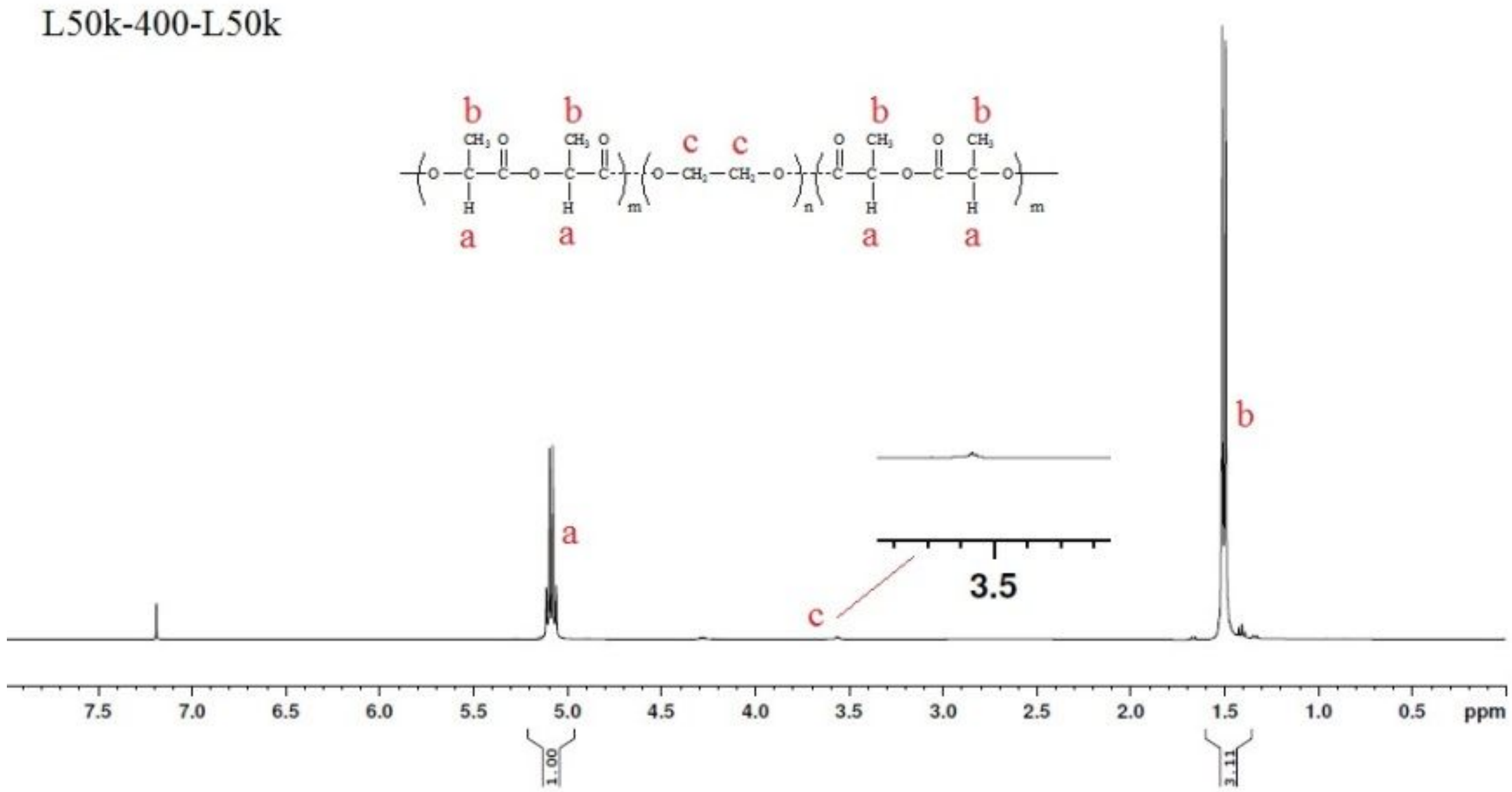

***Figure S3.** ^{1}H-NMR spectrum of L50K-400-L50K (PLLA–PEG400–PLLA triblock, target PLLA block Mn = 50,000 g/mol each) in $CDCl_3$ at 400 MHz.*

Peaks a, b (PLLA), peak c (PEG methylene, δ 3.65 ppm) and peak d (PEG–ester linkage, δ 4.25 ppm) all present, confirming triblock architecture. Due to the very low PEG400 mass fraction (WPEG ≈ 0.4%), peak c is small but reproducibly detected. Mn,NMR = 87,000 g/mol; Mn,GPC = 60,600 g/mol (PDI = 1.65). The GPC underestimation is consistent with compact PLA–PEG coil dimensions in THF [32].

S2.2.4. L100K-400-L100K

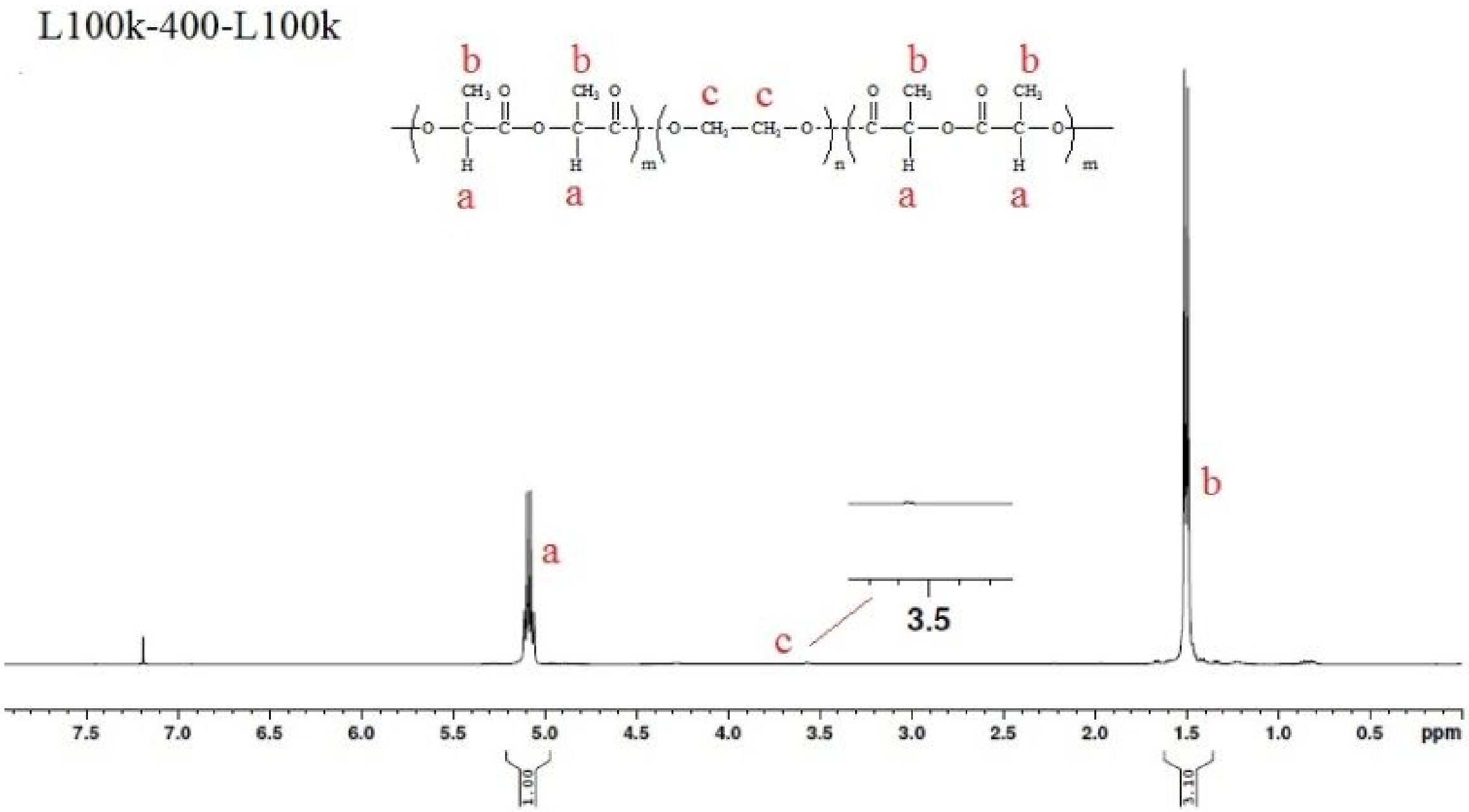

Figure S4. *¹H-NMR spectrum of L100K-400-L100K (PLLA–PEG400–PLLA triblock, target PLLA block Mn = 100,000 g/mol each) in $CDCl_3$ at 400 MHz.*

All five peaks confirmed. The c/a ratio is smaller than in L50K-400-L50K due to the higher PLLA block Mn at fixed PEG400 mass. Mn,NMR = 160,000 g/mol; Mn,GPC = 178,000 g/mol (PDI = 1.10). Close NMR/GPC agreement at the higher Mn confirms well-controlled synthesis.

S2.2.5. L50K-4k-L50K

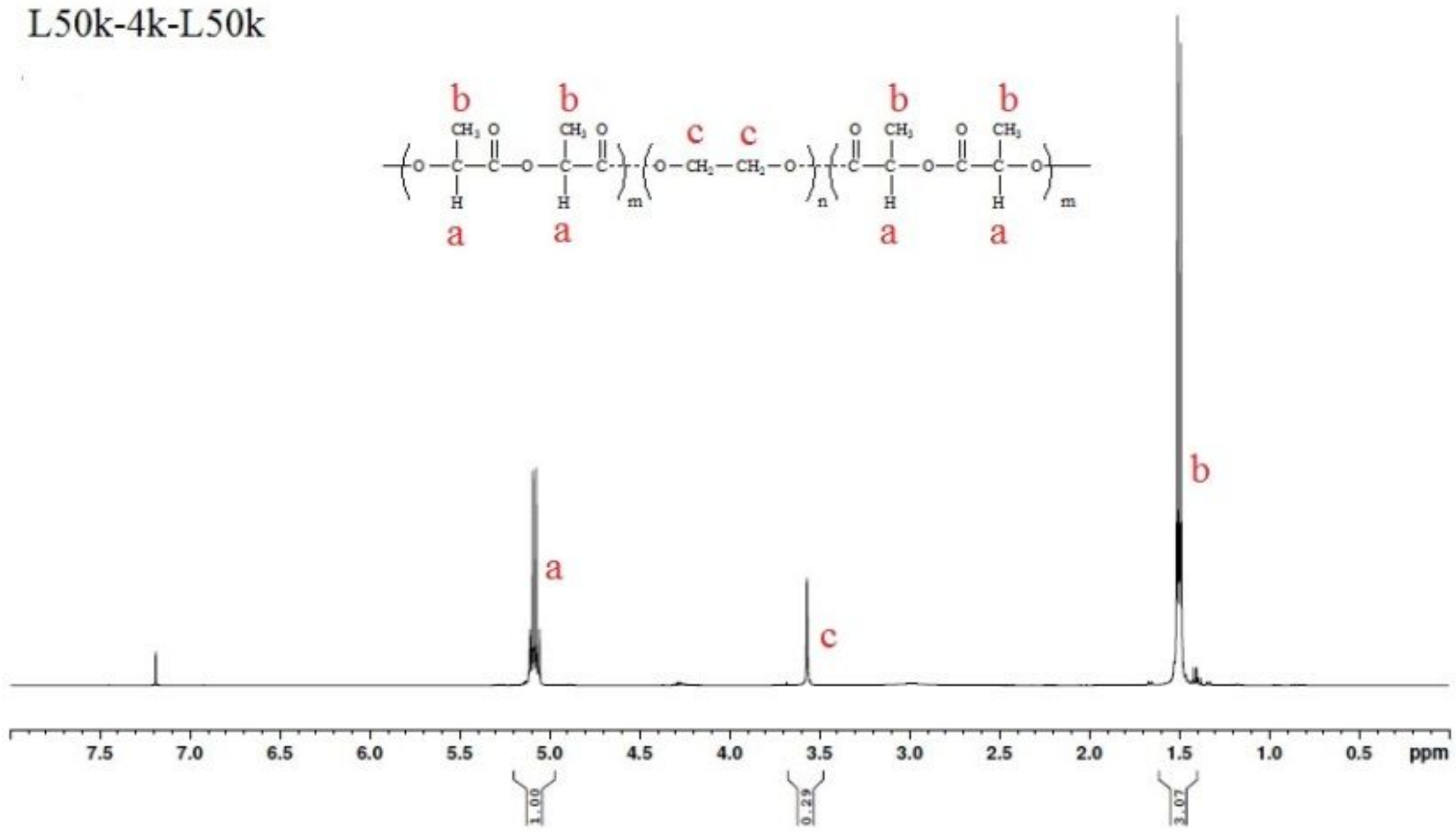

Figure S5. *^{1}H-NMR spectrum of L50K-4k-L50K (PLLA–PEG4k–PLLA triblock, target PLLA block Mn = 50,000 g/mol each) in $CDCl_3$ at 400 MHz.*

Peak c (δ 3.65 ppm) is clearly larger than in the PEG400 series, reflecting the higher PEG4k mass fraction (WPEG ≈ 3.8%). Peak d (δ 4.25 ppm) confirms covalent triblock connectivity. Mn,NMR = 74,000 g/mol; Mn,GPC = 48,000 g/mol (PDI = 1.40). The ~35% GPC underestimation relative to NMR is attributed to the compact coil architecture of PEG4k-containing triblocks in THF.

S2.2.6. L100K-4k-L100K

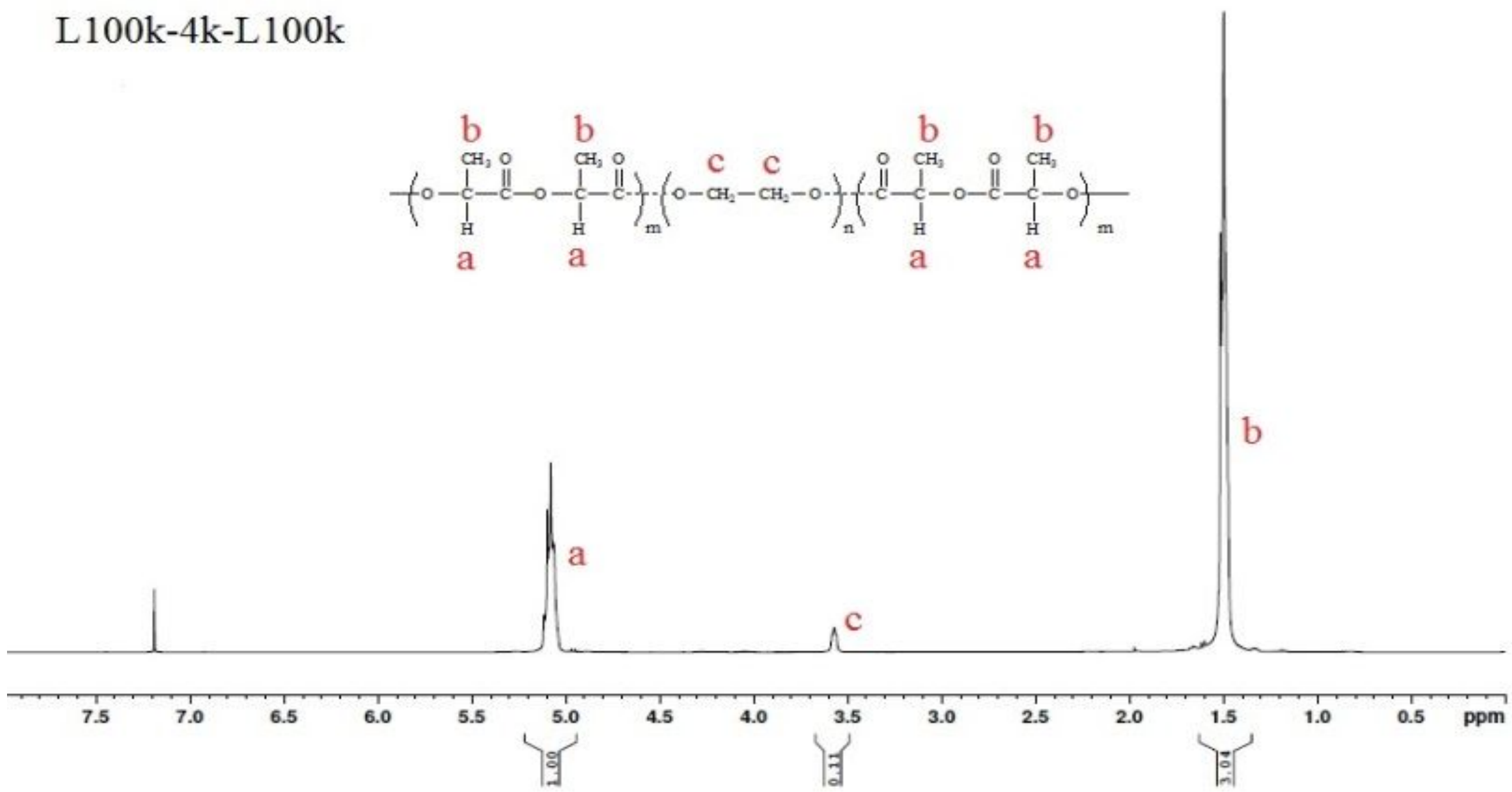

Figure S6. *^{1}H-NMR spectrum of L100K-4k-L100K (PLLA–PEG4k–PLLA triblock, target PLLA block Mn = 100,000 g/mol each) in $CDCl_3$ at 400 MHz.*

All five peaks clearly resolved. The c/a ratio is smaller than in L50K-4k-L50K because the larger PLLA blocks dominate the integral. Mn,NMR = 174,000 g/mol; Mn,GPC = 170,000 g/mol (PDI = 1.15). Excellent NMR/GPC agreement confirms well-controlled synthesis at this Mn.

S2.2.7. L50K-35k-L50K

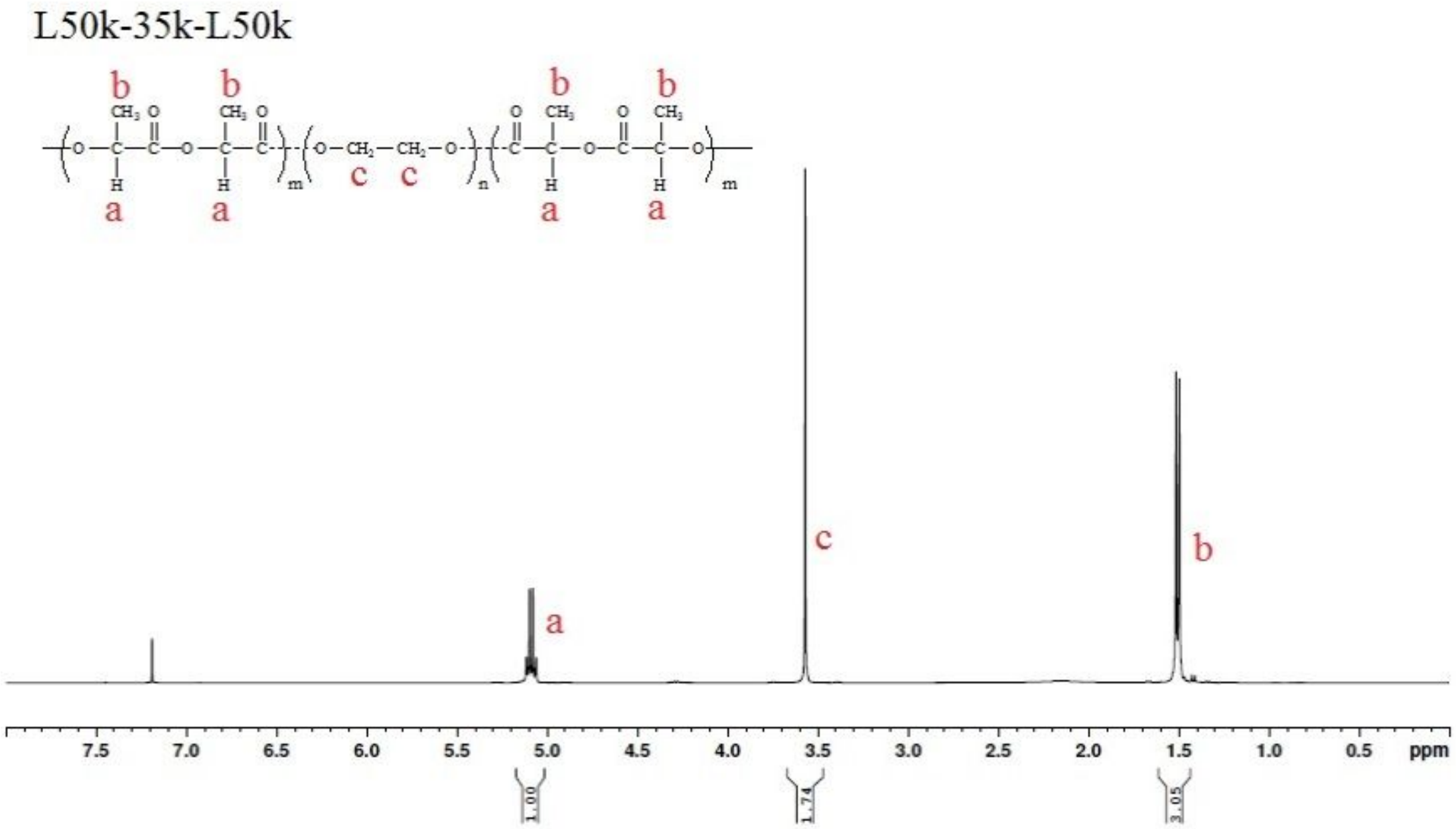

Figure S7. *¹H-NMR spectrum of L50K-35k-L50K (PLLA–PEG35k–PLLA triblock, target PLLA block Mn = 50,000 g/mol each) in $CDCl_3$ at 400 MHz.*

Peak c is the most prominent resonance in the spectrum, reflecting the substantial PEG35k mass fraction (WPEG ≈ 25.9%). Peak d (δ 4.25 ppm) is well resolved, confirming covalent block connectivity. Mn,NMR = 96,000 g/mol. Mn,GPC = 75,200 g/mol (PDI = 1.26); the ~28% GPC underestimation is the largest of all compositions and is attributed to the pronounced hydrodynamic volume compression of the compact PEG35k coil in THF, as documented by Xie et al. [26] and Dorgan et al. [32]. The WPLA correction (WPLA = 0.741) is therefore critical for this sample (see Table 3 of the main manuscript).

S2.2.8. L100K-35k-L100K

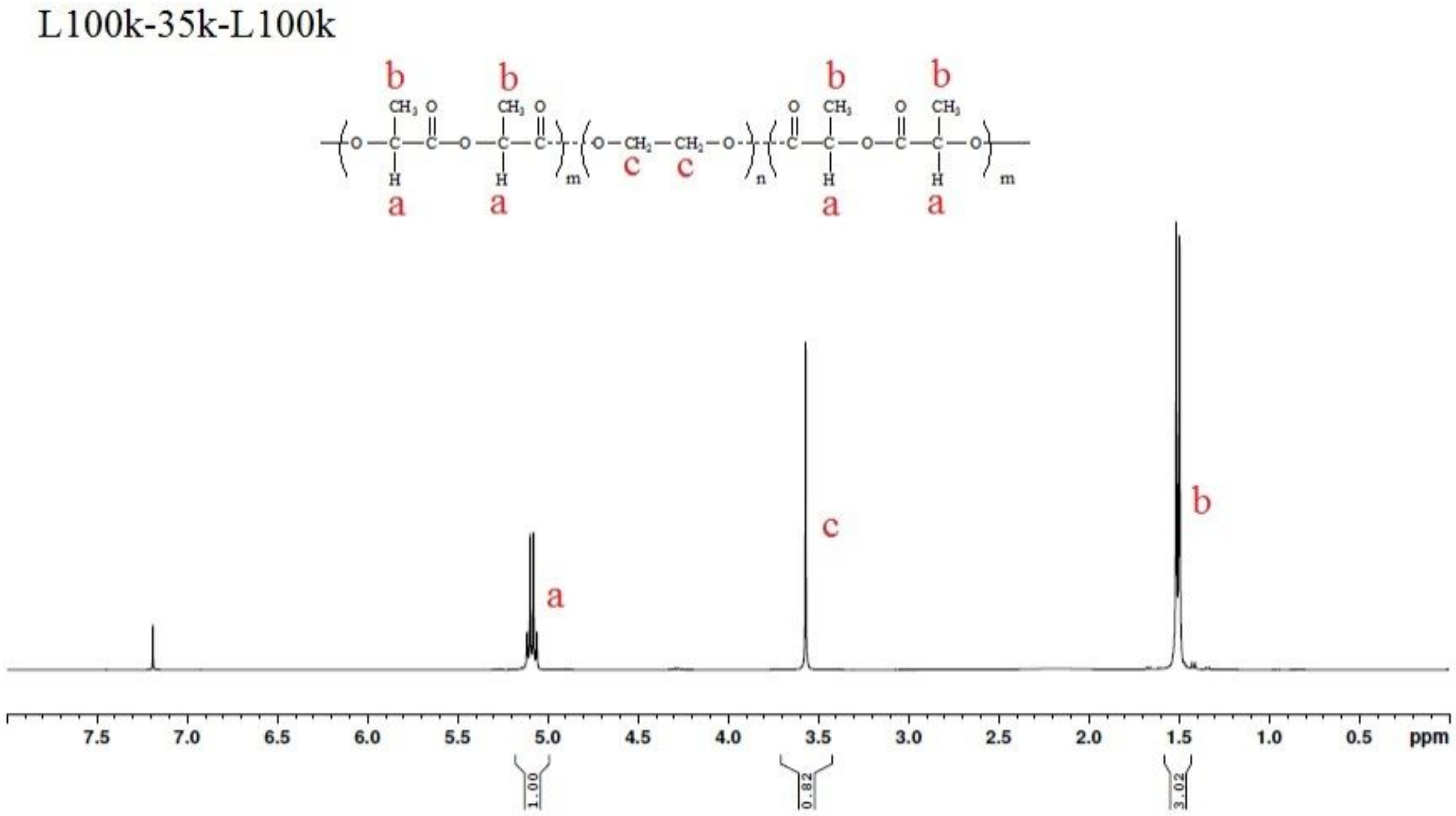

Figure S8. *¹H-NMR spectrum of L100K-35k-L100K (PLLA–PEG35k–PLLA triblock, target PLLA block Mn = 100,000 g/mol each) in $CDCl_3$ at 400 MHz.*

All five peaks are resolved. The c/a ratio is smaller than in L50K-35k-L50K because the doubled PLLA block Mn increases peak a relative to peak c at fixed PEG35k. Mn,NMR = 205,000 g/mol; Mn,GPC = 180,000 g/mol (PDI = 1.10). The consistently narrow PDI confirms well-controlled synthesis at all Mn targets.

Section S3: Solution-Cast Film Reference DSC Data

Table S1 presents DSC thermal properties of solution-cast films for all single-component polymers and SC blend compositions. These data serve as kinetic benchmarks for the injection-moulded specimens discussed in the main manuscript (Sections 3.3 and 3.5). Solution-cast films were prepared as described in Section 2.4 of the main manuscript (1:1 mass ratio, 0.5 g each in 10 mL chloroform, slow evaporation at ambient temperature over 12–24 h). The slow evaporation rate provides a near-equilibrium crystallisation opportunity compared to the rapid quench of injection moulding (~100–200°C s^{-1}).

Comparison of Table S1 with Table 3 of the main manuscript reveals that solution-cast films show consistently higher crystallinity than injection-moulded specimens. The crystallinity deficit ranges from ~7 pp (PEG35k specimens, which have enhanced crystallisation kinetics) to ~12 pp (neat PLLA). Importantly, PEG4k SC films show complete stereocomplexation (fSC = 100%) in the solution-cast form, while the

corresponding injection-moulded specimens retain residual homocrystals (fSC = 92.9–94.7%), directly quantifying the kinetic barrier imposed by rapid mold cooling that PEG35k uniquely overcomes (fSC = 100% in both modes).

Table S1. *DSC thermal properties of solution-cast films. WPLA-corrected crystallinity values. fSC from Eq. (3) of the main manuscript. For injection-moulded counterparts, see Table 3 of the main manuscript.*

Sample (Film)	Tg (°C)	Tc (°C)	ΔHc (J/g)	Tm1 (°C)	χhomo,corr (%)	Tm2 (°C)	χSC,corr (%)	fSC (%)
L100K	49	86	1.4	173	45.4	—	—	—
L200K	50	88	0.6	176	43.0	—	—	—
L50K-400-L50K	38	80	4.1	164	47.9	—	—	—
L100K-400-L100K	40	86	3.4	170	46.4	—	—	—
L50K-4k-L50K	38	84	1.6	170	48.2	—	—	—
L100K-4k-L100K	39	86	1.9	176	44.8	—	—	—
L50K-35k-L50K	35	97	9.4	170	49.1 (66.2[a])	—	—	—
L100K-35k-L100K	37	81	2.9	173	46.2 (62.3[a])	—	—	—
L100K/PDLA	55	—	—	171	3.1	219	~25.8	~89.3
L200K/PDLA	60	—	—	173	9.1	219	~16.5	~64.5
L50K-400-L50K/PDLA	62	—	—	164	2.6	219	~30.1	~92.0
L100K-400-L100K/PDLA	56	—	—	165	3.3	219	~27.2	~89.2
L50K-4k-L50K/PDLA	51	—	—	—	0	225	~34.6	100
L100K-4k-L100K/PDLA	53	—	—	—	0	224	~32.5	100
L50K-35k-L50K/PDLA	48	—	—	—	0	227	~40.6[b]	100
L100K-35k-L100K/PDLA	46	—	—	—	0	221	~37.8	100

[a] Values in parentheses are WPLA-corrected (WPLA = 0.741); uncorrected values shown first for direct comparison with injection-moulded data. [b] ΔHSC of L50K-35k-L50K/PDLA solution-cast film = 85.8 J/g (measured); χSC = 85.8 / (142 × 0.741) × 100 = 40.6% after WPLA correction.
